# Chitinase-3-like protein 1 depletion in glioma cells alters tumor microenvironment and normalizes neovasculature in human glioma xenografts

**DOI:** 10.1186/s12964-025-02636-8

**Published:** 2026-01-15

**Authors:** Salwador Cyranowski, Małgorzata Zawadzka, Mitrajit Ghosh, Bartosz Wojtas, Anna R. Malik, Katarzyna Poleszak, Kacper Waśniewski, Szymon Baluszek, Julian Swatler, Kamil Wojnicki, Bartłomiej Gielniewski, Beata Kaza, Agata Klejman, Hanna Łukasik, Bozena Kaminska

**Affiliations:** 1 Laboratory of Molecular Neurobiology, Nencki Institute of Experimental Biology, Polish Academy of Sciences, Warsaw, Poland; 2 Postgraduate School of Molecular Medicine, Medical University of Warsaw, Warsaw, Poland; 3 Laboratory of Neuromuscular Plasticity, Nencki Institute of Experimental Biology, Polish Academy of Sciences, Warsaw, Poland; 4 Laboratory of Sequencing, Nencki Institute of Experimental Biology, Nencki Institute of Experimental Biology, Polish Academy of Sciences, Warsaw, Poland; 5 Cellular Neurobiology Research Group, Faculty of Biology, University of Warsaw, Warsaw, Poland; 6 Laboratory of Cytometry, Nencki Institute of Experimental Biology, Polish Academy of Sciences, Warsaw, Poland; 7 Animal House, Nencki Institute of Experimental Biology, Polish Academy of Sciences, Warsaw, Poland

**Keywords:** CHI3L1, YKL-40, Glioblastoma, CRISPR editing, Extracellular matrix, Neoangiogenesis, Vasculature normalization

## Abstract

Chitinase-3-like protein 1 (CHI3L1) is a secreted, non-enzymatic glycoprotein that interacts with cell-surface and extracellular-matrix proteins, proteoglycans, and polysaccharides. Many studies reported the overexpression of CHI3L1 in various cancers, but its exact role in tumorigenesis/cancer progression remains elusive. We performed a comprehensive analysis of *CHI3L1* expression in public repositories including single-cell RNAseq datasets to determine the cellular source of *CHI3L1* expression in gliomas. The highest CHI3L1 expression was detected in glioblastoma (GBM), a high-grade diffusive brain tumor with dismal survival prognosis. CHI3L1 knockout (KO) in human U87-MG glioma cells grossly affected transcriptional profiles and in vitro invasiveness of these cells, and strongly reduced the growth of intracranial U87-MG tumors in athymic mice. CHI3L1 KO in glioma cells resulted in normalization of tumor vasculature. Co-culture of CHI3L1 KO glioma cells with astrocytes upregulated aquaporin 4 (AQP4) in p38/MAPK and IL-1β/NFκB-dependent manner. Diminished infiltration of glioma-associated myeloid cells in CHI3L1 KO tumors was associated with reduction in SPP1 expression in CHI3L1 KO cells. Altogether, we demonstrate that CHI3L1 depletion affects several mechanisms crucial for GBM progression, therefore its targeting represents a novel strategy to treat GBM patients.

**Supplementary Information:**

The online version contains supplementary material available at 10.1186/s12964-025-02636-8.

## Introduction

Chitinase-3-like protein 1 (CHI3L1, YKL-40) is a secreted non-enzymatic 40 kDa glycoprotein that binds proteins and carbohydrates, including chitin, heparin, and hyaluronic acid [1]. The ability of CHI3L1 to bind proteins and carbohydrates permits interactions with a spectrum of cell-surface and extracellular matrix proteins, proteoglycans, and polysaccharides. Several interacting partners of CHI3L1 play multifaceted roles in physiology and disease, making CHI3L1 signaling network difficult to apprehend. CHI3L1 interacts with IL13Rα2, CD44v3/IL13Rα2 complex, syndecan-1 and integrin α_V_β_5_, and signals through multiple pathways involved in allergic reactions, wound healing, inflammation resolution, epithelial-to-mesenchymal transition (EMT), tumor metastasis and angiogenesis [1–5].

CHI3L1 mRNA/protein levels are elevated in diseases characterized both by chronic inflammation and tissue remodeling such as cancer [6, 7]. Its principal source are activated macrophages, chondrocytes, neutrophils, and cancer cells [5]. Increased serum CHI3L1 concentration correlates with malignancy, poorer prognosis, and shorter overall survival in breast, colon, prostate, ovaries, brain, thyroid, lung, and liver cancer patients [8]. CHI3L1 affects the activity of matrix metalloproteinases (MMPs), influencing cell adhesion and migration, tissue remodeling, fibrosis, and tumorigenesis [3]. Recent studies implicate CHI3L1 in the regulation of immune checkpoint expression, immune checkpoint inhibitor efficacy and melanoma progression [9].

In the brain, *CHI3L1* upregulation occurs during neuroinflammation associated with many neurologic disorders, mostly in reactive astrocytes and macrophages but not in microglia [10, 11]. CHI3L1 protein was elevated in 65% cases of glioblastoma (GBM), the most common and lethal primary brain tumor with a median overall survival of 15 months [12, 13]. GBM is a highly diffusive tumor, accompanied by an intensive accumulation of myeloid cells that support tumor progression. *CHI3L1* overexpression in the Cancer Genome Atlas (TCGA) glioma dataset was identified as one of markers of the mesenchymal subtype, the most aggressive GBM subtype [14]. CHI3L1 serum levels were substantially elevated in numerous GBM patients and correlated positively with higher tumor grade and tumor burden [12]. Higher CHI3L1 expression was associated with poor responses to radiation, shorter time to progression and shorter overall survival [15].

A significant role of CHI3L1 in the development and progression of various cancers through promoting tumor cell invasion and metastasis have been documented but CHI3L1 affected pathways and contributing mechanisms are not fully understood in GBM. CHI3L1 affects endothelial tubulogenesis similarly to vascular endothelial growth factor (VEGF) [16] and increases VEGF expression by syndecan-1-integrin α_V_β_5_-pFAK signaling in human U87-MG glioma cells. Elevated CHI3L1 protein content was associated with elevated VEGF content in subcutaneous U87-MG and SNB-75 gliomas [17]. CHI3L1 counteracts the effects of a VEGF inhibitor bevacizumab in GBM, likely via vascular mimicry, production of angiogenic factors and modulation of pericyte coverage of blood vessels [18]. The exact role of CHI3L1 in GBM vascularization remains poorly understood. Astrocytic endfeet envelops blood vessels and contributes to the integrity of the blood–brain barrier by reinforcing endothelial junctions and regulating the exchange of substances between blood and brain tissue [19]. In addition, astrocytes are key players in the glymphatic system—a brain-wide drainage pathway analogous to the lymphatic system—where they facilitate the clearance of waste products and excess interstitial fluid from the perivascular space through aquaporin 4 (AQP4) water channels concentrated at their endfeet [20].

In this study we performed a comprehensive analysis of *CHI3L1* expression in TCGA and single-cell glioma datasets and glioma cell lines to pinpoint the cellular source of *CHI3L1* and resolve roles of this protein in GBM progression. We determined the effects of CHI3L1 knockout in human U87-MG glioma cells on transcriptomic patterns, tumor cell proliferation, migration, and microglia dependent invasion in co-cultures. We report reduced growth of intracranial CHI3L1 KO gliomas in nude mice, decreased infiltration of glioma associated myeloid cells, normalization of tumor vasculature and heightened AQP4 expression around blood vessels. The results indicate specific roles of CHI3L1 in glioma progression and underlying mechanisms.

## Materials and methods

### Public data

Analysis of public data was performed using R Statistical Software (version 4.2.2; R Foundation for Statistical Computing, Vienna, Austria). RNAseq data (FPKMs) were downloaded from the Ivy Glioblastoma Atlas Project [21] and normalized RNAseq and protein data from The Brain Protein Atlas (BPA) [22, 23]. Correlation of CHI31L1 mRNA and protein expression was computed in the BPA dataset, using Kendall correlation and von Waerden test followed by post-hoc U-Mann-Whitney tests to determine differences between tumor regions. Exact Fisher tests was performed for overlap of genes, whose expression correlated with CHI3L1, and the signature by Neftel et al. [24].

### Patient samples

We analyzed data from two cohorts: 76 glioma samples described in our previous study [25] and 31 glioma samples collected and analyzed by RNA sequencing in our previous study [26]. The collection of samples was approved by the Bioethics Committee of respective hospitals. RNAs from normal brains were purchased from: Agilent 540,005 (lot 0006127195, Female 66 years), Biochain R1234035-50 (lot B304105, Male 25 years), Human Brain Reference RNA AM6050 (lot 1207015, mix of RNA, male and female donors).

### Human cell lines and cell culture

Human glioblastoma cell lines U251, U87-MG-RFP (ATCC, Manassas, VA) and GBM patient-derived WG9 cell cultures [27] were maintained in Dulbecco’s modified Eagle’s medium (DMEM) with 10% fetal bovine serum (FBS) (Gibco, MD, USA) and antibiotics (100 U/mL penicillin, 100 µg/mL streptomycin). Normal human astrocytes (NHA; Lonza, Cologne, Germany) were cultured in dedicated Clonetics media (Lonza, Walkersville, MD, USA) in CO_2_/air (5%/95%) at 37 °C (Heraeus, Hanau, Germany). Cells were passaged when confluent up to 10x from the initial seeding. All cells used in the study including genome modified cells tested negative for *Mycoplasma*.

### RNA extraction and quantitative PCR (RT-qPCR)

Cells were washed with PBS and collected using a cell scraper and centrifugation. RNA was isolated using RNeasy Mini Kit (QIAGEN, USA) and reverse transcription was performed using SuperScript III Reverse Transcriptase (Invitrogen) on 500 ng RNA. Quantitative PCR was performed on 12.5 ng (for the detection of *CHI3L1* mRNA) or 50 ng (detection of *MMP2* and *IL1B* mRNA) of cDNA in duplicates using SYBR^®^ Green detection reagents and primers listed in Table 1.

**Table 1:** Primers for qPCR

Target mRNA	Sequence or reference
CHI3L1 human	qHsaCED0044484, BioRad
MMP2 human	forward 5′-GATGGCTTCCTCTGGTGCTC reverse 5′-TGGTGAACAGGGCTTCATGG
IL-1b human	Hs01555410_m1, ThermoFisher Scientific
GAPDH human	forward 5′- AGGGCTGCTTTTAACTCTGGT-3′ reverse 5′- CCCCACTTGATTTTGGAGGGA-3′
18 S human	forward 5′-CGGACATCTAAGGGCATCAACA-3′ reverse 5′-AACGAACGAGACTCTGGCATG-3′

### CRISPR/Cas9 genome editing

U87-MG-RFP and U251 human glioma cells (2 × 10^6^) were electroporated with 0.5 µg pCMV-Cas9-GFP plasmid and 2 sgRNAs targeting CHI3L1 (sgCHI3L1) (150 pmol each, Sigma, Munich, Germany) in SE electroporation buffer (100 µL) using a Nucleofactor 4DX according to manufacturer’s instructions (Lonza, Cologne, Germany). U251 cells were also transfected with control sgRNA (sgCTR) to provide a negative control for the cell line with CHI3L1 KO. Cells were then seeded in 20 mL standard culture media and maintained under standard culture conditions for 24 h before sorting. WG9 primary human glioma cells (1 × 10^6^) were transfected by lipofection using XtremeGENE 360 (Merck, Darmstadt, Germany) according to the manufacturer’s instructions with 5 µg pCMV-Cas9-GFP plasmid and the sgCHI3L1 and sgCTR mentioned above. After 6 h of lipofection, media were exchanged for fresh and then cells were allowed to grow for 24 h in standard culture media before sorting.

### Fluorescence-activated cell sorting and clonal selection

U87-MG-RFP and U251 cells were detached and sorted to 96-wells flat-bottom culture plates into 100 µL of 4.5 g glucose GlutaMAX™ DMEM supplemented with 15% FBS (Gibco, MD, USA) using BD FACSAria II cell sorter (BD Pharmingen). For U87-MG-RFP, gates for sorting were set for RFP^high^ and GFP^+^ events (Suppl. Figure 2a); for U251, gate was set for GFP^+^ events (Suppl. Figure 4a). WG9 cells were sorted in bulk using GFP signal (Suppl. Figure 5a) and seeded onto a 6-wells plate and allowed to grow for 72 h before reseeding. Next, cells were reseeded onto multiple 96-wells flat-bottom culture plates in a single-cell mode so that on average 1 cell was seeded onto 1 well. Media were changed every 3 days for a medium composed with 50% of a glioma-conditioned medium produced using U87-MG-RFP, U251 or WG9 (containing growth factors) and 50% fresh 4.5 g glucose GlutaMAX™ DMEM supplemented with 15% FBS. When colonies were established, cells were upscaled sequentially to 48-wells, 24-wells, 12-wells and 6-wells culture plates. All cell lines used were then tested for CHI3L1 expression by RT-qPCR and ELISA to select a clone with lowest CHI3L1 expression for further experiments. All cell lines were observed for deviations in morphology using light microscopy. Flow cytometry experiments were performed at the Laboratory of Cytometry, Nencki Institute of Experimental Biology.

### Enzyme-linked immunosorbent assay (ELISA)

To select U87-MG-RFP, U251 and WG9 CHI3L1 KO clones, cell culture supernatants were analyzed for the presence of human CHI3L1 using Human Chitinase 3-like 1 Quantikine ELISA Kit (R&D Systems, USA). Supernatants were centrifuged for 10 min at 300 × *g* to remove residual cells. In case of U87-MG-RFP, supernatant protein content of SPP1 was measured with Osteopontin Human ELISA Kit (Abcam, Cambridge, UK) and IL-1β with Human IL-1 beta/IL-1F2 ELISA Quantikine Kit (R&D Systems, USA). Tests were performed according to manufacturer’s protocol and measurements were acquired with a scanning multiwell spectrophotometer.

### RNA sequencing

RNA was isolated using the RNeasy kit (Qiagen) and RNA quality/yield was verified using Bioanalyzer 2100 (Agilent Technologies, Santa Clara, CA). mRNA libraries were prepared using KAPA Stranded mRNAseq Kit (Roche, Basel, Switzerland) according to the manufacturer’s protocol (KR0960-v6.17). mRNAs were enriched from 500 ng of total RNA using poly-T oligo-magnetic beads (Kapa Biosystems, MA, USA), fragmented, cDNA synthesized, and adapters were ligated. Loop structures were cut by USER enzyme (NEB, Ipswich, MA, USA). The obtained dsDNA fragments were amplified with NEB starters (Ipswich, MA, USA). Quality of libraries was determined using Bioanalyzer High Sensitivity dsDNA Kit (Agilent Technologies, Palo Alto, CA, USA) and concentrations measured using Quantus Fluorometer and QuantiFluor ONE Double Stranded DNA System (Promega, Madison, Wisconsin, USA). Libraries were sequenced on HiSeq 1500 (Illumina, San Diego, CA 92122 USA) on the rapid run flow cell with a paired-end settings (2 × 76 base pairs). Fastq files were aligned to hg38 human reference genome with STAR program [28], and reads were counted to genes using feature Counts algorithm SUBREAD package [29]. Gene counts were normalized, and differential analysis was performed using the Wald test from the DESeq2 package [30]; differentially expressed genes (DEG) were defined as observations with FDR corrected p-value below 0.05. REACTOME pathway analyses [31] were performed using R package clusterProfiler [32].

### Intracranial glioma implantation

The study was approved by the First Local Ethics Committee in Warsaw, Poland (1123/2020). Male mice (Foxn1^nu^, Janvier, France) were housed in individually ventilated cages, fed standard chow, and kept under standard day/night conditions. Ten weeks old mice were anesthetized with isoflurane anesthesia (Temsega, Pessac, France). Appropriate analgesics were used for the procedure. WT or CHI3L1 KO U87-MG-RFP glioma cells (5 × 10^4^ cells in 1 µl of DMEM) were implanted aseptically into the right striatum using a stereotactic apparatus and 1 µL syringe with a 26-gauge needle (Stoelting Co., Wood Dale, USA).

### Magnetic resonance imaging

The animals were anesthetized with 1.5–2.5% isoflurane (Baxter, Deerfield, IL, USA) in oxygen. Animal heads were scanned with 7 T BioSpec 70/30 MR system (Bruker, Ettlingen, Germany) equipped with Avance III console and actively shielded gradient system B-GA 20 S (amplitude 200 mT/m). Structural transverse MR images covering the whole brain were acquired with T2-weighted TurboRARE (TR/TEeff = 7000/30 ms, RARE factor 4, spatial resolution = 86 μm×86 μm×350 μm, 42 slices, no gaps, number of averages 4, scan time ~ 23 min).

### Collecting brain tissues and blood samples

The animals were anesthetized 21 days post-implantation with injections of ketamine (160 mg/kg) and xylazine (20 mg/kg) and perfused transcardially with an ice-cold PBS and 4% paraformaldehyde (PFA). Beforehand, the right atrium was perforated and 500 µL of blood was collected. Brains were removed and fixed in 4% PFA for 48 at 4 °C. Then, brains were placed in 30% sucrose in PBS at 4 °C. Finally, brains were frozen in Tissue Freezing Medium (Leica, Germany) and cut into 12–50 μm thick coronal sections and stored. Collected blood was kept for 2 h at RT until coagulated and centrifuged for 10 min at 3,000 x *g*. Blood serum was supplemented with proteinase inhibitor PMSF (phenylmethylsulfonyl fluoride, 1 mM, Sigma, Munich, Germany) and stored at −80 °C. Blood sera were analyzed using Human/Mouse Chitinase 3-like 1 Quantikine ELISA Kits (R&D Systems, USA).

### Fluorescence microscopy

For vWF, IBA1, GFAP, AQP4, SPP1, CD206 immunostaining, brain sections were thawed, dried for 2 h in RT, rehydrated in PBS for 15 min and washed 3x in 0.05 M TBS (Tris-buffered saline, Sigma, Munich, Germany). Then, sections were incubated with 10% donkey serum (DS) with 0.1% Triton X-100 (in TBS) for 2 h at RT and stained overnight or for 48 h (SPP1) at 4 °C with primary antibodies listed in Table 2. Secondary antibodies were incubated for 2 h at RT, sections were washed and mounted with Vectashield Vibrance^®^ Antifade Mounting Medium with DAPI (Vector Labs, US). Additionally, for CD206 staining, brain sections were heated to 100 °C for antigen retrieval before staining procedure. For GFAP/AQP4 co-staining, CD31 and laminin staining, brain sections were thawed and dried for 5 min at 37 °C temperature and then kept in chilled methanol in −20 °C for 30 min. Next, sections were washed 3x for 5 min in PBST (0.1% Triton X-100 in PBS), blocked with 3% DS in 0.4% Triton X-100 in PBS for 2 h at RT, stained overnight with primary antibodies at 4 °C followed by incubation with secondary antibody for 2 h at RT. Sections were washed 3x in PBST for 5 min, once in PBS for 5 min, followed by washing in ultrapure water for 5 min and mounted with Vectashield Vibrance^®^ Antifade Mounting Medium with DAPI (Vector Labs, US). In negative controls primary antibody was omitted. Images were captured using Zeiss Axio Imager M2 microscope. Image processing and quantification was performed using ImageJ software (NIH). Confocal images were taken using Zeiss LSM 780 or Zeiss LSM800 Airyscan microscope at the Laboratory of Imaging Tissue Structure and Function, Nencki Institute. The images shown in the Fig. 5e are 3D renderings of z stacks: 639 × 639 μm and at least 40 μm of z stack (z steps 3.5 μm) using Bitplane Imaris Image Analysis software.

**Table 2:** Antibodies and their source

Antibody	Working dilution	Company
goat anti-IBA1	1:500	ab5076, Abcam, UK
rabbit anti-IBA1	1:1000	019–19741, Wako, Japan
rabbit anti-von Willebrand factor	1:1000	A008229-2, Agilent, USA
rabbit anti-AQP4	1:200	ab125049, Abcam, UK
rabbit anti-GFAP	1:200	Z033429-2, Agilent USA
rat anti-CD31	1:100	#NB600-1475, Novus Biologicals, USA
rabbit anti-laminin	1:50	ab11575, Abcam, UK
goat anti-SPP1	1:100	AF1433, R&D, USA
rabbit anti-CD206	1:500	ab64693, Abcam, UK
rabbit anti-Ki67	1:1000	ab92353, Abcam, UK
donkey anti-goat Alexa Fluor 488	1:1000	A-11,055, Invitrogen, USA
donkey anti-rabbit Alexa Fluor 647	1:1000	A-31,573, Invitrogen, USA
donkey anti-rabbit Alexa Fluor 488	1:1000	A-21,206, Invitrogen, USA
donkey anti-rat Alexa Fluor 488	1:500	A-21,208, Invitrogen, USA

### Matrigel invasion assay

Cell culture inserts (12 μm pore size, Millpore, Tullagreen, Ireland) were coated with 1 mg/mL Growth Factor Reduced Matrigel Matrix (BD Biosciences, CA, USA) in DMEM and dried at 37 °C for 5 h. Human SV40 immortalized microglial cells (SV40) were cultured in PriCoat T25 flasks in Prigrow III medium (Applied Biological Materials, Richmond, Canada) supplemented with 10% FBS (Gibco, MD, USA). One day prior to the co-culture, SV40 cells were seeded at 4 × 10^4^ on a 12-wells plate in dedicated medium, glioma cells were seeded at 4 × 10^4^ on matrigel-covered membrane in medium dedicated for microglial cells with 2% FBS. Inserts were transferred to a 24-wells plate with or without microglial cells. After 18 h, cells invading through the matrigel were fixed with 95% methanol and stained with DAPI (4′,6- Diamidino-2-Phenylindole; 0.01 mg/ml, Sigma, Munich, Germany). The membranes were cut out and cells were visualized using fluorescence microscope (Leica DM4000B, ×10 objective). Cell nuclei were counted from five independent fields per slide using ImageJ software.

### Gelatin zymography

U87-MG cells (5 × 10^5^ cells per well) were seeded onto 24-wells plate in 1 mL DMEM with 10% FBS (Gibco, MD, USA) and antibiotics. After 24 h, media were replaced with DMEM, and cells were incubated for 24 h. The culture media were collected, centrifuged for 10 min at 300 × *g* and subjected to gelatin zymography as previously described [33]. Culture media with FBS were used as a positive control.

### Primary astrocyte cultures

Primary astrocyte cultures were prepared from newborn (P1) C57BL/6J mice as described previously [34]. In brief, blood vessels and meninges were removed from cortical tissue which was next detached with trypsin and dissociated in presence of DNAse (Gibco, MD, USA). Cells were plated on poly-L-lysine-coated (Sigma, Munich, Germany) flasks and cultured in primary cultures dedicated medium: DMEM 4.5 g glucose GlutaMAX™ supplemented with 10% FBS (Gibco, MD, USA) and antibiotics (100 U/mL penicillin, 100 µg/mL streptomycin). After 48 h, cells were washed with PBS to remove cellular debris. Next, cultures were shaken extensively (at DIV10 and DIV14) to remove microglia and oligodendrocyte precursor cells. Then, astrocytes were seeded on 6-wells plate and transduced with SV40 expressing lentiviral particles.

### Lentivirus production

HEK 293 T cells were transfected with the following plasmids: pBABE-puro SV40 LT, pUMVC (packaging), and pCMV-VSV-G (envelope) (all from Addgene, MA, USA) with use of PEI reagent (Polysciences, PA, USA) in DMEM without serum (Merck, Darmstadt, Germany). Plasmids used were isolated from bacterial cultures with Syngen Endofree Plasmid MAXI Kit + Filter [Syngen, Wroclaw, Poland]. Produced lentiviral particles were purified from the cell culture medium.

### Astrocyte transduction and selection

Astrocytes (7 × 10^5^ cells/well on a 6-wells plate) were transduced with lentiviral particles using MOI = 50. Two days after transduction, the medium was replaced with the fresh astrocyte culture medium. Three and nine days after transduction, the medium was replaced with the astrocyte culture medium containing 2 µg/ml puromycin. Colonies of cells forming during selection were re-plated to new plates and cultured further in the presence of puromycin until they reached confluence. Next, the cells were cultured in the astrocyte culture medium.

### GCM treatment of astrocytes

U87-MG-RFP cells were seeded onto 100 mm dish at the density of 1 × 10^6^ in a standard medium. Following 24 h, the medium was exchanged for a medium dedicated for primary cultures. Following another 24 h, the glioma conditioned medium (GCM) was collected and centrifuged for 10 min at 300 × *g* to get rid of floating cells. Supernatants were collected and stored with PMSF (1 mM, Sigma, Munich, Germany) at 4 °C for up to 7 days. Immortalized primary astrocytes were treated with GCM for 24 h at the density of 3 × 10^5^ on a 6-wells plate. For IL-1β neutralizing antibody treatment, immortalized astrocytes were seeded onto 12-wells plate at the density of 1 × 10^5^ in the dedicated medium. Following 24 h, media were swapped for GCM produced with CHI3L1 KO cells and 2 µg/mL anti-IL-1β antibody was added (R&D Systems, USA). Then, astrocytes were washed, lysed and the cell lysates stored with 1 mM PMSF at −80 °C until processed further for Western blotting.

### Glioma–astrocyte co-cultures and p38/MAPK inhibitor or IL-1β neutralizing antibody treatment

U87-MG-RFP cells were seeded onto 0.4 μm cell culture inserts (Corning, AZ, USA) at the density of 7.5 × 10^4^ in a standard medium, while immortalized astrocytes were seeded onto 12-wells plate at the density of 1 × 10^5^ in the dedicated medium. Following 24 h, glioma cell containing inserts were transferred to the wells with astrocytes. For p38 inhibitor treatment, 1 mL dedicated astrocyte medium was added to the wells with 20 µM p38/MAPK inhibitor (MedChemExpress LLC, NJ, USA) or DMSO (Sigma, Munich, Germany) followed by adding 300 µL of the dedicated astrocyte medium to the inserts. After 24 h, astrocytes were rinsed, lysed and the cell lysates were stored with 1 µM PMSF at −80 °C until processed further for Western blotting.

### SDS-PAGE and Western blotting

Cells were lysed in the buffer containing phosphatase and protease inhibitors (20 mM Tris HCl, pH 6.8, 137 mM sodium chloride, 25 mM β-glycerophosphate, 2 mM sodium pyrophosphate, 2 mM EDTA, 1 mM sodium orthovanadate, 1% Triton X-100, 10% glycerol, 5 µg/mL leupeptin, 5 µg/mL aprotinin, 2 mM benzamidine, 0.5 mM DTT, 1 mM PMSF). The protein concentration was determined with Bradford Protei Assay (Thermo Fisher Scientific, MA, USA). Protein extracts (12 µg) were separated on SDS-PAGE using precast gradient 4–15% gels (Biorad, Hercules CA, USA) before electrophoretic transfer onto a nitrocellulose membrane (Amersham Biosciences, Germany) as described [35]. After blocking with 5% non-fat milk in TBS-T (Tris-buffered saline pH 7.6/0.15% Tween 20) the membranes were incubated with primary antibody recognizing p-p38 (Cell Signaling Technology, MA, USA), AQP4 (ProteinTech, IL, USA for GCM and anti-IL-1β treatment and Abcam, Cambridge, UK for p38/MAPK inhibitor experiments) and NFκB (Cell Signaling Technology, MA, USA) diluted in TBS-T, milk or 5% BSA (Sigma, Munich, Germany) overnight at 4 °C and then with horseradish peroxidase-conjugated anti-rabbit IgG (Vector Laboratories, CA, USA) for 1 h at RT. Immunocomplexes were visualized by using SuperSignal West Pico PLUS Chemiluminescent Substrate (Thermo Fisher Scientific, MA, USA). The membranes were stripped and re-probed with horseradish peroxidase-conjugated anti-β-Actin (Sigma, Munich, Germany) or anti-GAPDH (ProteinTech, IL, USA) antibody to verify total protein loading. For densitometry, band intensities were measured for 3 experiments with following software: Image Lab (Biorad) or Image Studio Lite (LI-COR Biosciences) and ImageJ (NIH).

## Results

### CHI3L1 is highly overexpressed in glioblastoma and is associated with the mesenchymal subtype markers

**Fig. 1:**
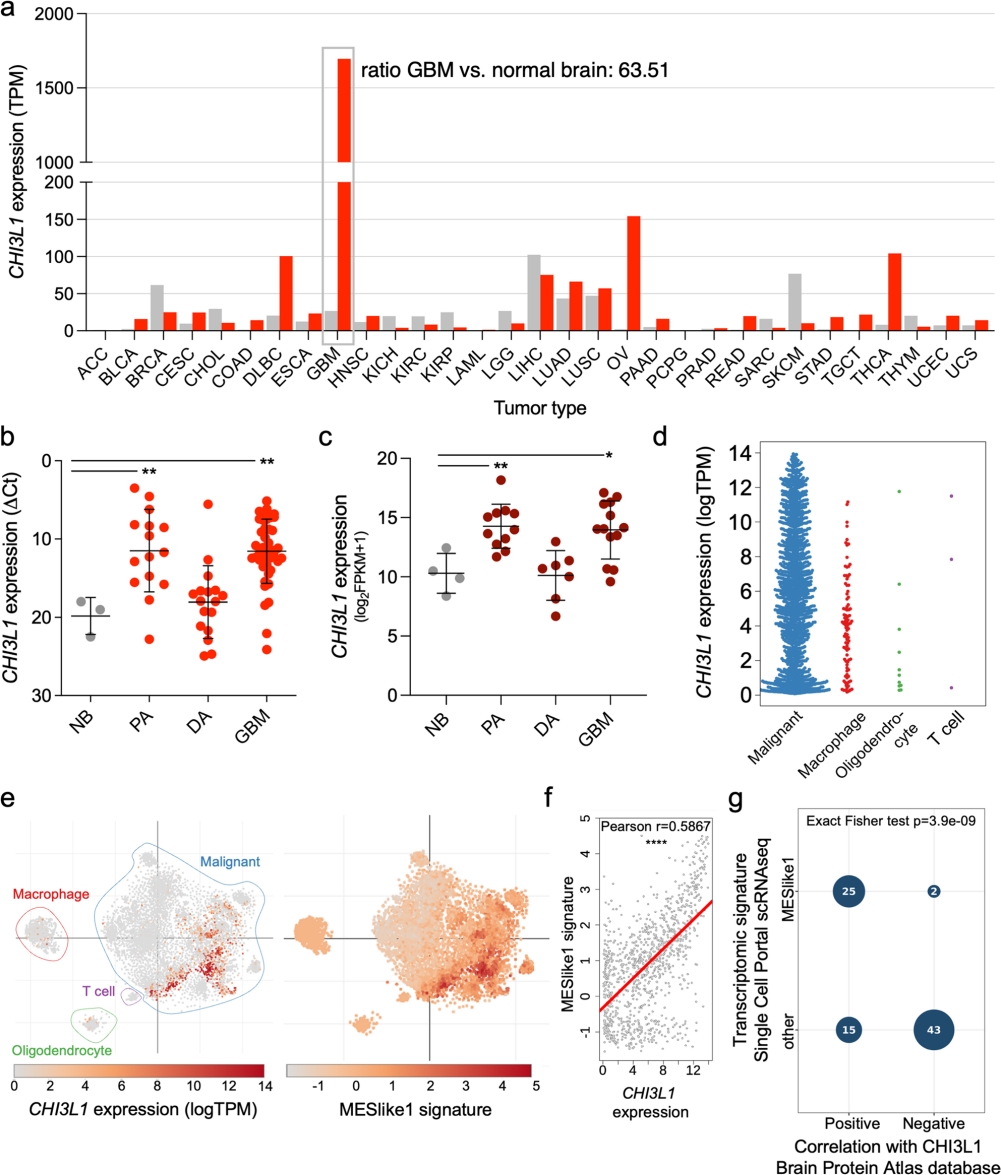
*CHI3L1* is highly overexpressed in glioblastoma and is associated with the mesenchymal subtype. **a** Bar plot of *CHI3L1* expression profile across various tumor samples and paired normal tissues from the GEPIA database. TPM, transcripts per million. Full names of tumor types are in Suppl. Fig. 1. **b** RT-qPCR gene expression analysis of *CHI3L1* in patient derived samples (cohort 1), data is presented as mean ± SD; *n* = 3 for NB, normal brain; *n* = 15 for PA, pilocytic astrocytoma; *n* = 17 for DA, diffusive astrocytoma; *n* = 44 for GBM, glioblastoma. Statistical analysis by one-way ANOVA, Dunnet’s multiple comparison test, * *P* ≤ 0.05, ** *P* ≤ 0.01. **c** Bulk-RNAseq gene expression analysis of *CHI3L1* in 31 patient derived samples (cohort 2); *n* = 4 for NB, *n* = 11 for PA, *n* = 7 for DA, *n* = 13 for GBM. **d-f** *CHI3L1* expression analysis in a dataset of 28 *IDH* wild-type human GBM tumors at single-cell resolution (Single Cell Portal, Broad Institute). **d** Beeswarm plot of *CHI3L1* expression in malignant, stromal, and immune cells in GBM. **e** Left: tSNE plot showing a distribution of *CHI3L1* expression across GBMs at single-cell resolution. Right: tSNE plot representing the mesenchymal (MESlike1) signature in the dataset. **f** Scatter plot representing a positive correlation between the mesenchymal signature and *CHI3L1* expression. Linear regression line in red. **** *P* ≤ 0.0001. **g** Correlation analysis between genes associated with mesenchymal signature in the Single Cell Portal and proteins correlating with CHI3L1 in the Brain Protein Atlas database. Exact Fisher test. Numbers in circles indicate the number of genes/proteins

We determined *CHI3L1* expression in glioma bulk and single-cell RNA sequencing (scRNAseq) datasets. Comparison of *CHI3L1* expression in various tumors in the GEPIA database [36] shows that *CHI3L1* expression is highest in GBM among 32 cancer types (Fig. 1a) with 63.5 times higher expression in GBM vs. normal brain (Suppl. Figure 1). The quantitative PCR (RT-qPCR) analysis of *CHI3L1* expression in the samples from 76 gliomas [25] shows upregulated expression of *CHI3L1* in malignant (GBM) and benign gliomas (pilocytic astrocytoma) compared to normal brain tissue (Fig. 1b). Similarly, high *CHI3L1* expression was detected in the bulk-RNAseq dataset of 31 gliomas [26] (Fig. 1c). The analysis of the published scRNAseq dataset of 28 *IDH1* wild-type GBMs shows that *CHI3L1* was expressed predominantly by malignant cells, with some expression in glioma-associated macrophages (Fig. 1d-e). Interestingly, *CHI3L1* expression positively correlated with the mesenchymal signature (MESlike1), occurring in the aggressive transcriptional subtype of GBM (Fig. 1e-f). We cross-checked the genes listed in the MESlike1 signature with the proteins correlated with CHI3L1 in the Brain Protein Atlas database [22, 23]. Out of 27 genes assigned to the MESlike1 signature, 25 correlated positively with CHI3L1 at a protein level in GBMs (Fig. 1g).

### CHI3L1-depleted human glioma cells show altered expression of genes involved in extracellular matrix reorganization and cell adhesion

**Fig. 2:**
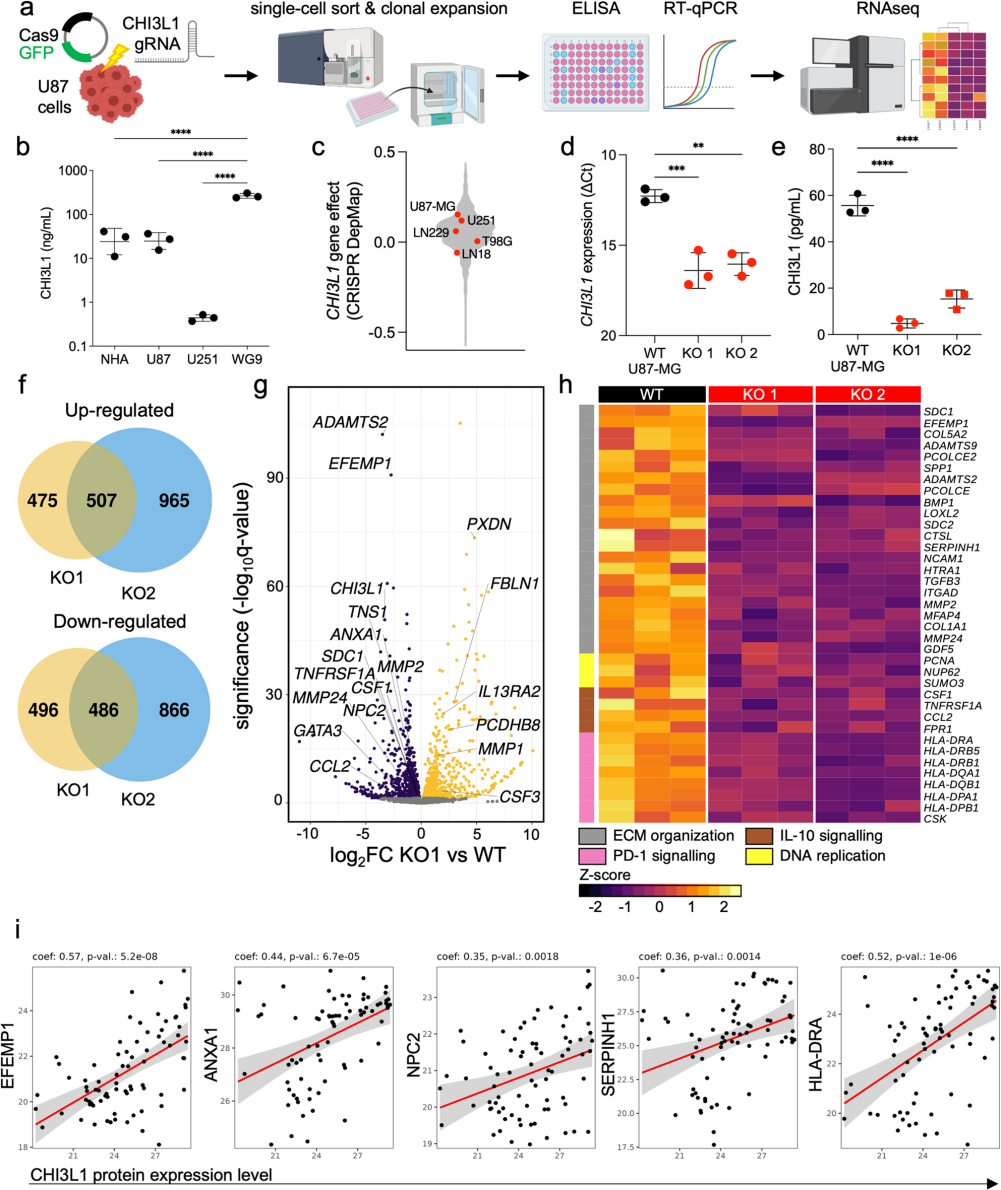
Knock-out of CHI3L1 in U87-MG glioma cells results in gross changes in the expression of genes involved in ECM re-organization and immunosuppression. **a** Experimental pipeline of generating CHI3L1 knock-out in U87-MG-RFP (U87) human glioblastoma cells using CRISPR/Cas9 technology. **b** Assessment of CHI3L1 expression on protein level in normal human astrocytes (NHA), two human glioma cell lines (U251 and U87-MG) and one human primary GBM cells (WG9); mean ± SD is presented; statistical analysis by one-way ANOVA, Tukey’s multiple comparison test; **** *P* ≤ 0.0001. **c** Cancer Dependency Map (DepMap, Broad Institute) plot of “gene effect” of tested human glioma cell lines. The higher the gene effect, the lower the impact of CRISPR-mediated knock-out of CHI3L1 on the viability of the cells. **d-e** Validation of CHI3L1 knock-out in U87 cells at mRNA (d) and protein level (e) in three independent passages; mean ± SD is presented; statistical analysis by unpaired t-test; ** *P* ≤ 0.01 (d); one-sample t-test with hypothetical value of 100% (e). ** *P* ≤ 0.01. **f-h** Comparative results of RNA sequencing between WT and CHI3L1 KO U87-MG cells. **f** Venn diagrams of differentially expressed genes (DEGs) for up- and down-regulated (upper and lower panel, respectively) genes between two CHI3L1 KO clones as compared to WT control. **g** Volcano plot of DEGs in the CHI3L1 KO clone 1 (KO1) compared to WT control. **h** Z-score heatmap of the REACTOME pathway enrichment analysis for WT, CHI3L1 KO1 and KO2 cells. Gene clusters indicated with color bars to the left. **i**. Scatter plots of protein expression from the Brain Protein Atlas study representing positive correlation between CHI3L1 and EFEMP1, ANXA1, NPC2, SERPINH1 and HLA-DRA proteins

To evaluate the effect of CHI3L1 absence, CRISPR/Cas9 genome editing was applied and human glioma cells depleted of CHI3L1 (CHI3L1 KO) were generated (Fig. 2a). U87-MG cells exhibited higher CHI3L1 protein/mRNA content compared to U251 and were selected for in vivo experiments (Fig. 2b, Suppl. Fig. 2e). While WG9 cells showed highest CHI3L1 protein/mRNA content, they failed to establish tumors in recipient mice (data not shown). We used the Cancer Dependency Map portal [37, 38] and found U87-MG cells to have the highest gene effect (a negative parameter predicting the effect of gene knock-out on cell viability) for CHI3L1 depletion among the commonly used human glioma cell lines (Fig. 2c).

U87-MG-RFP cells were transfected with a commercial Cas9-GFP plasmid and validated guide RNAs specific for two sites in the *CHI3L1* gene. Transfected cells (GFP+, Suppl. Fig. 2a) were sorted individually and expanded clonally. Multiple independent clones were subjected to RT-qPCR and ELISA to evaluate efficacy of knockout (Suppl. Fig. 2b-c). Two clones with the lowest CHI3L1 expression (KO1 and 2) were utilized further (Fig. 2d-e). We repeated the process for U251 and WG9 cells to obtain multiple cell culture models for CHI3L1 depletion (Suppl. Fig. 4a and 5a).

We performed RNAseq on wild-type (WT) and two U87-MG CHI3L1 KO clones (KO1 and KO2) and defined differentially expressed genes (DEGs) as differentially expressed between WT and both KO clones with FDR corrected *p-*value < 0.05. A Venn diagram shows numbers of commonly up- or down-regulated genes among DEGs in KO1 and KO2 clones compared to WT cells (Fig. 2f). Around 50% of DEGs were affected in both KO cell lines. A Volcano plot shows DEGs in KO1 cells (Fig. 2g) and Reactome pathway enrichment analysis reveals that many genes down-regulated in CHI3L1 KO clones belong to ECM organization, migration, and invasion pathways (*MMP2*, *MMP24*, *BMP1*, *PCOLCE*, *PCOLCE2*, *COL5A2*, *COL1A1*, *ADAMTS2*, *ADAMTS9*, *EFEMP1*, *TGFB3*) (Fig. 2h).

Genes highly up-regulated in CHI3L1 KO cells included genes associated with the formation and re-organization of ECM and cell-to-cell adhesion (*FBLN1*, *PXDN*, *MMP1*, *PCDHB8*) (Fig. 2g). Genes involved in PD-1 and IL-10 signaling were downregulated in CHI3L1 KO cells. *CSF1* encoding macrophage colony stimulating factor or *TGFB3* encoding transforming growth factor 3 were consistently downregulated in both clones (Fig. 2h). Some of the genes determined as mesenchymal-associated in single-cell database [24]: *ANXA1*, *EFEMP1*, *SPP1*, *NPC2*, *TNFRSF1A* were most down-regulated in CHI3L1 KO compared to WT cells (Fig. 2g-h).

We cross-checked DEGs in CHI3L1 KO cells with the Brain Protein Atlas study [22, 23] and found a positive correlation between CHI3L1 and proteins encoded by genes down-regulated in CHI3L1 KO cells (EFEMP1, ANXA1, NPC2, SERPINH1 and HLA-DRA) (Fig. 2i).

Altogether, CHI3L1 KO cells downregulated genes involved in ECM reorganization, invasion, immunosuppression and mesenchymal subtype.

### Glioblastoma cells depleted of CHI3L1 form smaller tumors in recipient mice

**Fig. 3:**
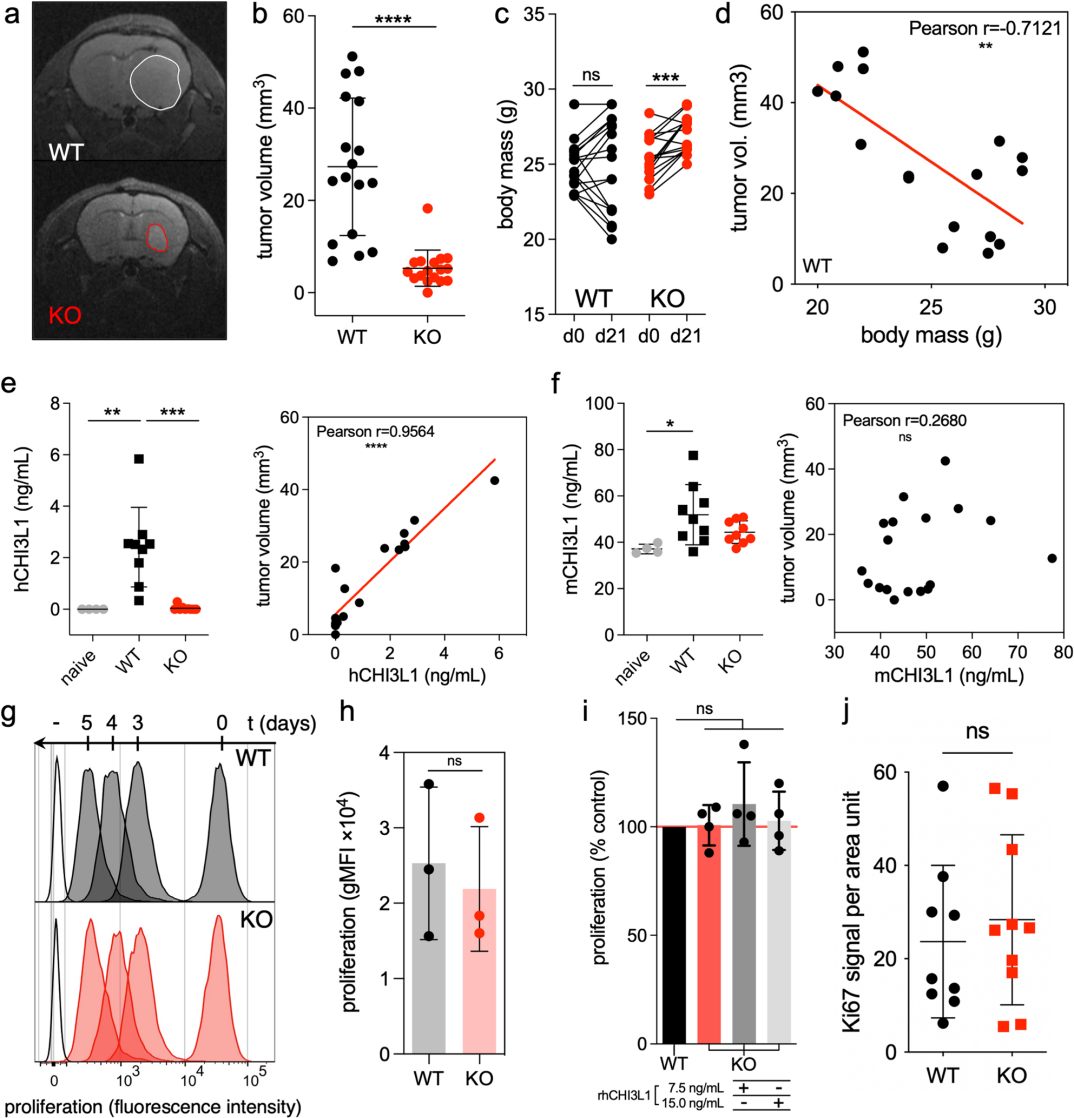
Depletion of CHI3L1 in U87-MG human glioblastoma cells reduces tumor growth in recipient mice. **a**-**b** Measurement of tumor growth by magnetic resonance imaging (MRI) 21 days post orthotopic implantation of glioma cells to athymic mice. **a** Representative head MRI scans showing tumor size reduction in CHI3L1 KO tumors compared to WT tumors. **b** Quantification of tumor volume measurement; mean ± SD, *n* = 17 per group; mean of WT = 27.3 mm^3^, mean of KO = 5.3 mm^3^; statistical analysis by parametric, unpaired t-test; **** *P* ≤ 0.0001. **c** Body mass comparison of recipient mice between day 0 and day 21 post implantation in WT and CHI3L1 KO groups; *n* = 17 per group; parametric, paired t-test; *** *P* ≤ 0.001. **d** Scatter plot of tumor volume versus corresponding body mass in WT group showing a negative correlation between the two parameters. **e-f** Measurement of human and mouse CHI3L1 concentration, respectively, in blood serum of tumor-bearing mice and a correlative analysis with the tumor volume; mean ± SD is presented, *n* = 4 for naïve, *n* = 9 for other groups; parametric, unpaired t-test; * *P* ≤ 0.05, ** *P* ≤ 0.01, *** *P* ≤ 0.001; linear regression line in red. **g-h** Proliferation analysis of U87-MG cells. **g** Protein dye-based proliferation assay. Histogram of fluorescent dye signal at time points 0, 3, 4 and 5 days of cell culture with the dye. Unstained control is designated ‘-’. **h** Quantification of fluorescent dye signal after 72 h of cell culture with the dye; mean ± SD is presented; *n* = 3, parametric unpaired t-test. **i** Quantification of cell proliferation with a BrdU proliferation assay using DNA-intercalating agent; mean ± SD is presented; *n* = 4, one-way ANOVA with Dunn’s multiple comparison test. Adding recombinant human CHI3L1 to WT and KO cells does not affect cell proliferation. The concentration of 7.5 ng/mL corresponds to the concentration of CHI3L1 produced by cultured WT cells. **j** Quantification of immunofluorescent staining for Ki67, a mitosis/proliferation marker, of WT or CHI3L1 KO tumor sections; mean ± SD is presented; parametric unpaired t-test

CHI3L1 KO glioma cells implanted intracranially into Foxn1^nu^ mice developed significantly smaller tumors compared to WT controls as demonstrated by magnetic resonance imaging (MRI) 21 days post-implantation (Fig. 3a-b). Mice in CHI3L1 KO group gained weight during tumor growth, which is an indicator of a lesser tumor burden in this group (Fig. 3c). Tumor volume and body mass of tumor-bearing animals were negatively correlated (Fig. 3d).

Human CHI3L1 (hCHI3L1) was detected in blood serum of mice bearing WT tumors and its concentration dropped substantially in mice bearing CHI3L1 KO tumors (Fig. 3e, left). hCHI3L1 serum concentration positively correlated with tumor volume in the WT group (Fig. 3e, right). Mouse CHI3L1 was detectable in the serum of naive mice and increased in mice bearing WT tumors but not CHI3L1 KO tumors (Fig. 3f, left). mCHI3L1 concentration did not correlate with tumor volume (Fig. 3f, right).

Importantly, diminished tumor growth in KO group was not due to reduced cell proliferation, as WT and CHI3L1 KO U87-MG cells had comparable proliferation rate in vitro (Fig. 3g-h). Adding the recombinant hCHI3L1 to culture media of CHI3L1 KO glioma cells did not increase CHI3L1 KO cell proliferation (Fig. 3i). Thus, extracellular CHI3L1 does not control glioblastoma proliferation in vitro. The lack of difference in proliferation rate between WT and KO cells was confirmed in vivo with Ki67 staining of tumor sections (Fig. 3j, Suppl. Fig. 3e). Notably, CRISPR/Cas9 manipulation did not affect the proliferation rate of U87-MG cells as confirmed with sgCTR clones (Suppl. Fig. 2d).

### Glioma-derived CHI3L1 enhances invasion and myeloid cell infiltration into the tumor

**Fig. 4:**
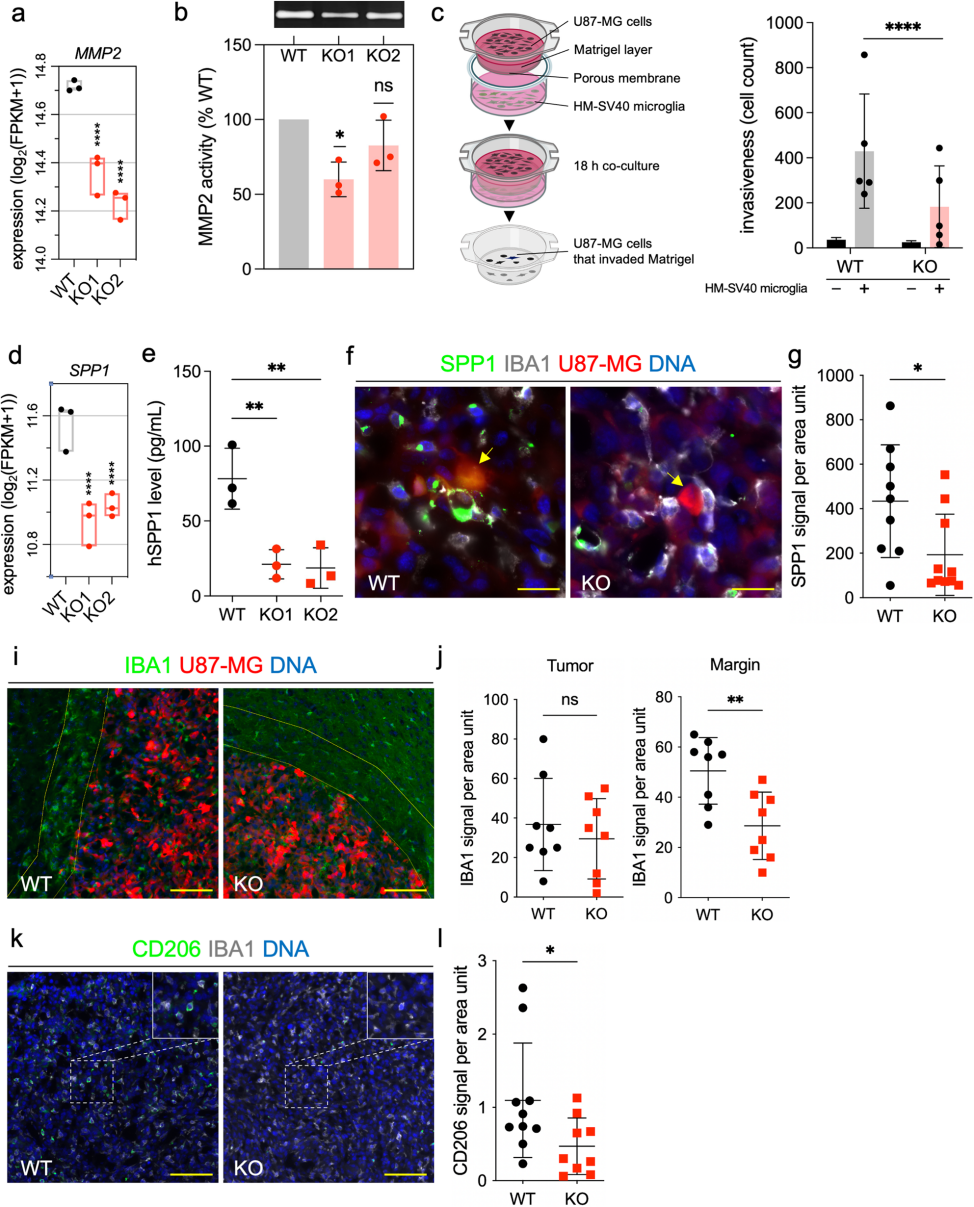
Depletion of CHI3L1 decreases invasion of cultured glioma cells and myeloid cell infiltration in experimental gliomas in vivo. **a** Boxplot of *MMP2* expression in CHI3L1 KO clones compared to WT control in the RNA-seq dataset; **** Padj ≤ 0.0001. **b** The gelatinolytic MMP2 activity in the U87-conditioned medium measured using gelatin zymography. Upper panel shows representative bands on the electrophoretic gel corresponding to the MMP2 gelatinase activity. Lower panel shows quantification of three biological repeats; data are shown as mean ± SD; statistical analysis with one-sample t-test with hypothetical value of 100%; * *P* ≤ 0.05. **c** Matrigel invasion assay for WT and CHI3L1 KO cells: scheme (left) and bar plot of results (right). Black bars represent baseline invasiveness of glioma cells; grey and red dots/bars represent invasiveness of WT and CHI3L1 KO cells when co-cultured with HM-SV40 human microglial cells; mean ± SD is presented; chi-squared test; odds ratio = 2.37; **** *P* ≤ 0.0001. OR stands for odds ratio. **d** Boxplot of *SPP1* expression in CHI3L1 KO cells compared to WT controls in the RNA-seq database; **** Padj ≤ 0.0001. **e** Measurement of SPP1 concentration in U87-conditioned media from CHI3L1 KO cells compared to WT control cells. Cytokine concentration was normalized to the cell lysate proteins; unpaired, parametric t-test; ** *P* ≤ 0.01. **f** Immunofluorescent staining of SPP1 (green) and a pan-myeloid cell marker IBA1 (light gray). Scale bar represents 24 μm. Arrows indicate U87 glioma cells with and without SPP1 signal. **g** Quantification of SPP1 immunostaining in tumor-bearing brain sections shown in f; unpaired, parametric t-test; * *P* ≤ 0.05. **i** Immunofluorescent staining of IBA1 (green). Scale bar represents 150 μm. Yellow lines indicate a 200 μm wide ROI around the tumor, denoting the tumor margin. **j** Quantification of IBA1 immunostaining in tumor-bearing brain sections shown in i, within the tumor (left) and the tumor margin (right); unpaired, parametric t-test; ** *P* ≤ 0.01. **k** Immunostaining of CD206 (green) and IBA1 (light gray). Scale bar represents 150 μm. Enlarged regions of interest are presented in the upper right corner. l. Quantification of CD206 staining in tumor-bearing brain sections shown in k; unpaired, parametric t-test; * *P* ≤ 0.05

CHI3L1 KO cells showed lower expression of *MMP2*, coding for metalloproteinase 2 than parental cells (Fig. 4a). We performed gelatin zymography and found a significantly lower gelatinolytic activity in conditioned media from CHI3L1 KO1 glioma cells compared to WT cells (Fig. 4b). We and others had demonstrated that glioma invasion is strongly enhanced by microglial cells [35, 39, 40]. Invasion of WT and CHI3L1 KO (KO1) glioma cells in the presence/absence of human HM-SV40 microglial cells was determined with a matrigel assay. We found reduced invasion of CHI3L1 KO cells in microglia presence (Fig. 4c). We confirmed reduced *MMP2* gene expression and decreased invasiveness in other cell lines with CHI3L1 KO (Suppl. Fig. 4e-f, 5d-e).

Brain resident microglia and monocyte-derived macrophages accumulate and are reprogrammed by GBM to support tumor progression, immunosuppression, and resistance to therapy [41, 42]. One of the reprograming factors in GBM is SPP1 (secreted phosphoprotein 1, osteopontin) [39]. The expression of *SPP1* was significantly down-regulated in CHI3L1 KO glioma cells both at mRNA and protein level (Fig. 4d and e, respectively). We also confirmed this finding in vivo by the immunostaining of SPP1 on tumor sections. Glioma cells in CHI3L1 KO tumors exhibited less SPP1 staining compared to WT group (Fig. 4f-g). We determined the infiltration of myeloid cells in intracranial gliomas by staining for IBA1, a pan-myeloid cell marker. Although there was no difference in IBA1 signal within the tumor area, we found the density of IBA1 + cells significantly decreased in the margin of CHI3L1 KO tumors compared to WT tumors (Fig. 4i-j). Immunostaining for CD206, a well-established marker of immunosuppressive macrophages in glioblastoma [43], revealed less CD206 + myeloid cells in CHI3L1 KO tumors compared to the WT group (Fig. k-l), indicating an altered phenotype despite no difference in myeloid cell number.

### Depletion of glioma-derived CHI3L1 results in normalization of tumor vasculature

**Fig. 5:**
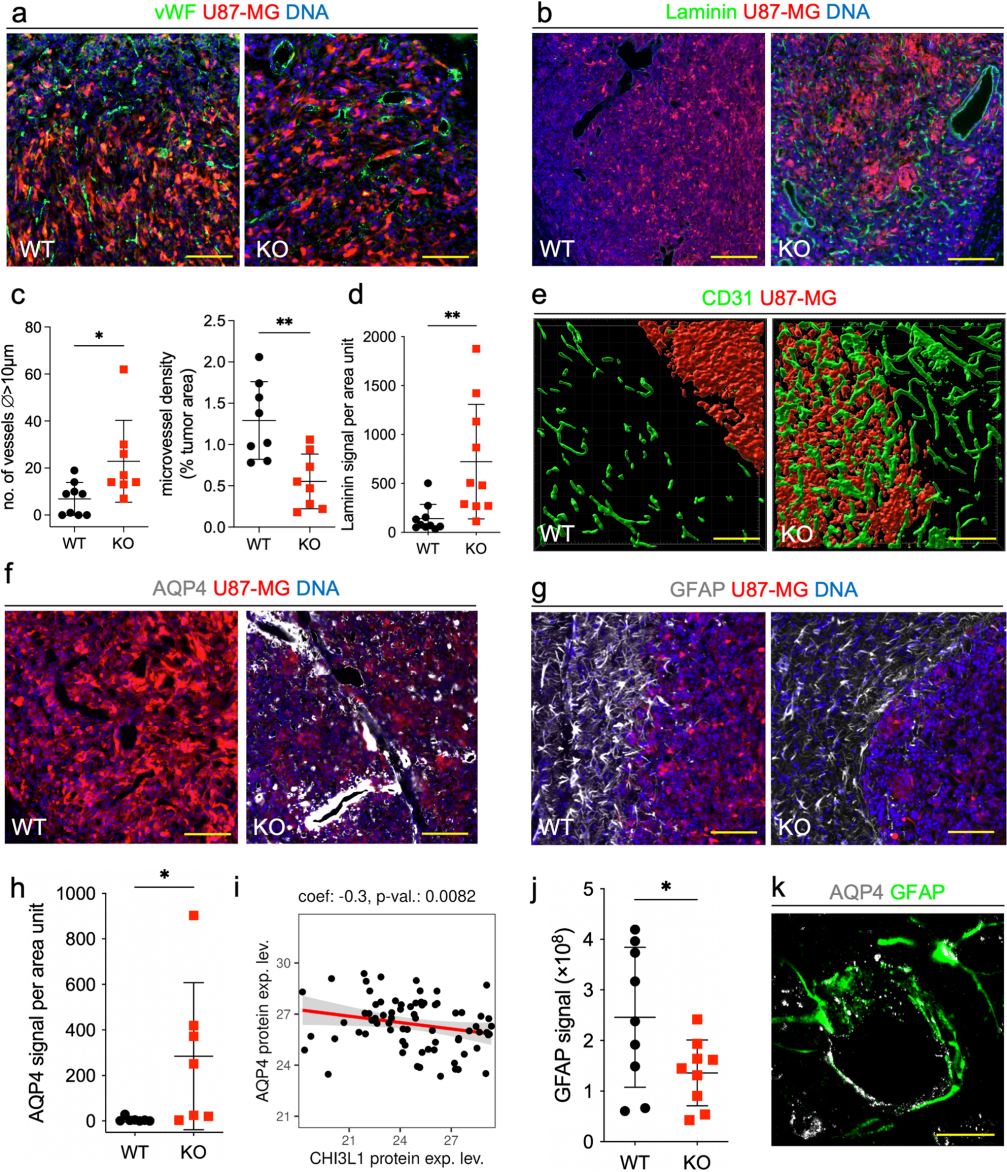
CHI3L1 depleted tumors show normalization of the vascular network and perivascular space. **a**-**g** Immunofluorescent staining for the von Willebrand Factor (vWF), an activated endothelium marker, in green (**a**); laminin, an epithelium/endothelium basement membrane marker, in green (**b**); endothelium marker CD31, in green (**e**); aquaporin 4 (AQP4), an astrocytic water channel in light gray (**f**); and glial fibrillar acidic protein (GFAP), an astrocytic marker in light gray (**g**). Scale bar represents 150 μm (**a**, **e**, **f**, **g**) or 300 μm (**b**). For CD31, 3D rendering performed with Imaris software. In all images U87-MG glioma cells are shown in red and DNA (DAPI staining) is shown in blue. **c** Quantification of non-capillary vessels (⌀ >10 μm) (left) and microvessel density (right) based on vWF staining shown in (a). **d** Quantification of laminin signal based on laminin staining in (b). **h** Quantification of AQP4 signal based on AQP4 staining shown in (f). **i** Linear regression analysis of CHI3L1 and AQP4 protein expression in the Brain Protein Atlas: proteomic data from patient GBM samples. **j** Quantification of GFAP signal based on GFAP staining shown in (**i**). For all plots, mean ± SD is shown; unpaired, parametric t-test; * *P* ≤ 0.05, ** *P* ≤ 0.01. **k** Double staining of AQP4 (in light gray) and GFAP (in green) at confocal microscope. Astrocytic endfeet around a blood vessel are captured. Scale bar represents 50 μm

GBM is one of the most highly vascularized solid tumors and it is characterized by an extensive proliferation of microvessels [44, 45], which leads to a high microvessel density (MVD). We compared the vasculature of WT and CHI3L1 KO U87-MG tumors by staining for von Willebrand Factor (vWF), an activated endothelium marker (Fig. 5a). The gross vasculature differed considerably in WT and CHI3L1 KO tumors; with more numerous non-capillary vessels (Fig. 5c left) and lesser MVD in the latter (Fig. 5c right). The number of vessels and MVD parameter did not correlate with the tumor volume (Suppl. Fig. 3a-b), suggesting that the increase in non-capillary vessels in CHI3L1 KO tumors was independent of tumor volume but resulted from CHI3L1 depletion instead.

We examined the structure of the blood vessel walls by staining tumor sections for laminin, an endothelium basement membrane marker (Fig. 5b). We observed that in CHI3L1 KO gliomas blood vessels were continuously lined with laminin in contrast to WT tumors. Laminin immunopositivity significantly increased in CHI3L1 KO gliomas vs. WT (Fig. 5d). The integrity of morphologically abnormal vessels was assessed using endothelium CD31 staining and 3D rendering. CD31 + vessels formed continuous tubes in CHI3L1 KO tumors in comparison to those in WT group (Fig. 5e). These findings show that in the absence of tumor-derived CHI3L1, blood vessels are better structured and likely more functional.

Coverage of blood vessels by astrocytic endfeet contributes to the blood-brain barrier by sealing endothelial cell junctions and controlling the transport of substances from blood [19]. Astrocytes participate in creating a network of CNS drainage similar to lymphatic system called glymphatic system, where they clear waste and excess of extracellular fluid from the perivascular space *via* a water channel aquaporin 4 (AQP4) [20, 46, 47], localized at the astrocytic endfeet. The expression of AQP4 was low in WT U87-MG gliomas but considerably increased in CHI3L1 KO gliomas (Fig. 5f and h). A linear regression analysis for CHI3L1 and AQP4 protein content in the BPA human tumors database shows a negative correlation between the two proteins in GBM (Fig. 5i). Reduced staining for glial fibrillary acidic protein (GFAP) demonstrates that astrocytes were less activated in CHI3L1 KO tumors in comparison to WT controls (Fig. 5g and j). Aquaporin 4 was localized at the GFAP + cell protrusions covering blood vessels as demonstrated with double staining for AQP4 and GFAP (Fig. 5k). We propose that tumor-derived CHI3L1 promotes deregulation of the vascular network in gliomas by increasing the number of endothelial sprouts, altering the structure of vascular walls, and decreasing the coverage of blood vessels by astrocytes (Fig. 7).

### **C**HI3L1 KO-associated AQP4 upregulation in astrocytes is mediated by p38/MAPK and IL-1β/NFκB pathways

**Fig. 6:**
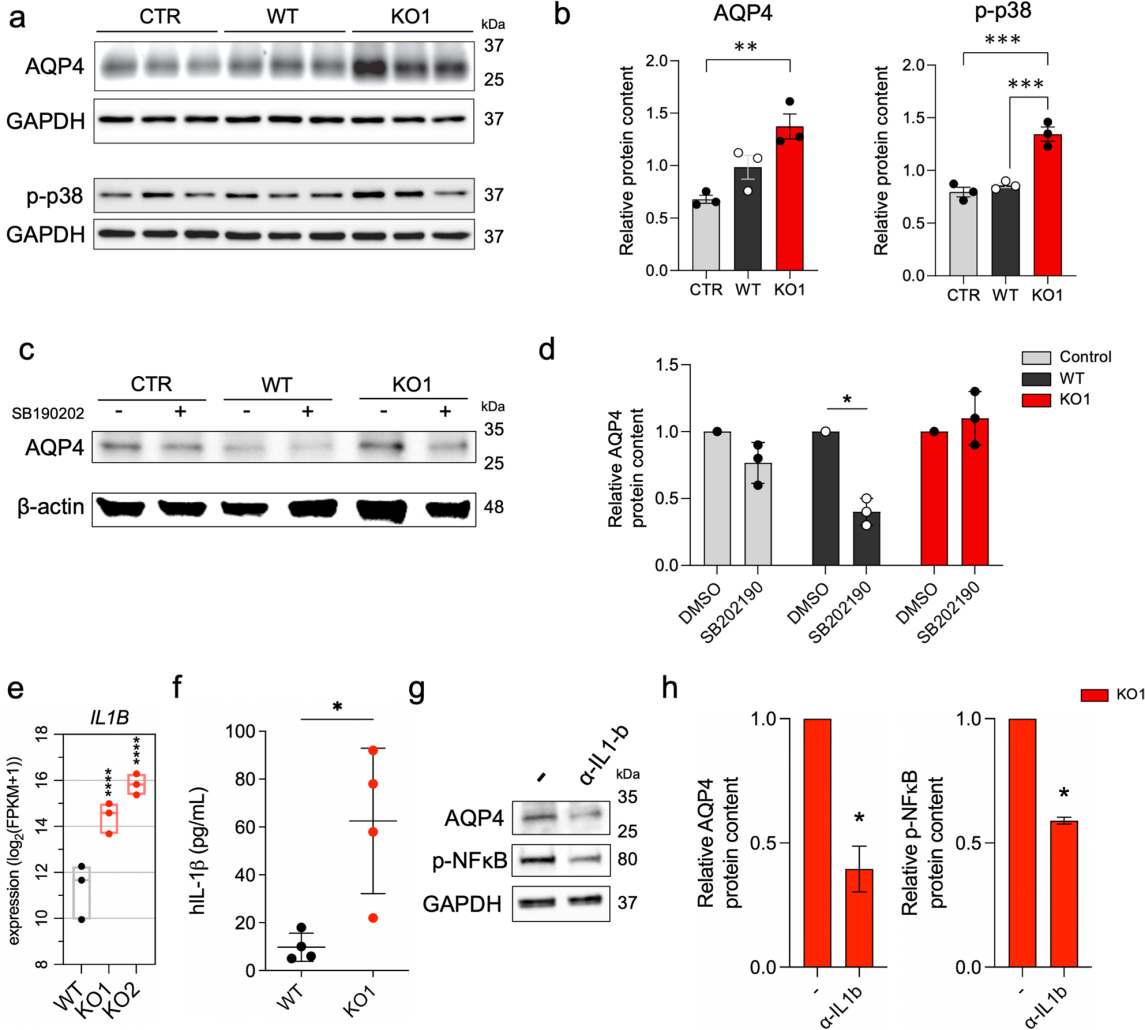
CHI3L1 KO-associated upregulation of AQP4 in astrocytes is mediated by p38/MAPK pathway. **a**-**b** Western blot analysis of protein content for AQP4 and p-p38 in immortalized mouse astrocytes after 24 h exposure to a control medium (CTR) and GCM from WT and CHI3L1 KO (KO1) cells. **a**. Representative blots for one experiment with 3 technical replicates for each condition. **b** Quantification for 3 biological repetitions shown in a, mean ± SD is shown, * *P* ≤ 0.05, ** *P* ≤ 0.01, *** *P* ≤ 0.001. **c-d** Western blot analysis of AQP4 protein content in immortalized mouse astrocytes after 48 h co-culture with WT and CHI3L1 KO cells with p38 inhibitor, SB190202 (20 µM). Representative blots and quantification from 3 biological repetitions, mean ± SD is shown, * *P* ≤ 0.05. **e** Boxplot of *IL1B* expression in CHI3L1 KO cells compared to WT controls in the RNA-seq database; **** Padj ≤ 0.0001. **f** Measurement of human IL-1β concentration in U87-conditioned media from CHI3L1 KO cells (KO1) compared to WT control cells; unpaired t-test, * *P* ≤ 0.05. **g-h** Western blot analysis of protein content for AQP4 and p-NFκB in immortalized mouse astrocytes after 24 h exposure to a GCM from CHI3L1 KO (KO1) cells and IL-1β neutralizing antibody (α-IL-1β). **g** Representative blots for one experiment. **h** Quantification for 3 biological repetitions. Fold change to non-treated condition shown. Paired t-test; mean ± SD is shown. ** *P* ≤ 0.01

AQP4 plays a role in maintaining fluid traffic and physiological intracranial pressure and its expression is tightly controlled. AQP4 expression is regulated by two mechanisms: either transcriptionally or by more rapid subcellular relocation of already present protein [48]. AQP4 transcription is regulated by the AP-1 transcription factor (among others), which is under the control of p38/MAPK pathway and downstream of IL-1β signaling [49, 50]. We had obtained SV40-immortalized mouse astrocyte cultures and examined the protein content of AQP4 and p-p38 (phosphorylated p38) in those astrocytes 24 h after stimulation with GCM collected from CHI3L1 WT and KO glioma cells. The protein content of AQP4 was significantly higher in astrocytes stimulated with GCM from CHI3L1 KO cells compared to control astrocytes (Fig. 6a and b left). The protein content of AQP4 was higher in astrocytes exposed to CHI3L1 KO GCM compared to WT GCM; however, this difference did not reach statistical significance. We observed a substantial increase in p-p38 protein content in astrocytes cultured in CHI3L1-depleted GCM over control and WT GCM conditions (Fig. 6a and b right).

To study the role of p38/MAPK pathway in GCM-driven expression of AQP4 in astrocytes, we used a commercially available p38 inhibitor (SB190202). Immortalized mouse astrocytes were co-cultured with WT or CHI3L1 KO glioma cells in the presence of SB190202 or DMSO (vehicle control). We observed a profound decrease in the AQP4 protein content in astrocytes co-cultured with WT cells after p38 inhibition (Fig. 6c-d). Interestingly, this decrease did not occur in astrocytes co-cultured with CHI3L1 KO cells. This demonstrates that the knockout of CHI3L1 in glioma cells causes astrocytes to retain the ability to express AQP4 independently of the p38/MAPK pathway.

IL-1β has been reported as a driver of AQP4 expression in murine astrocytes via NFκB [51]. We hypothesized that IL-1β expressed by CHI3L1 KO cells could induce AQP4 expression in astrocytes bypassing the p38/MAPK pathway. We assessed U87-MG, U251 and WG9 cells for IL-1β expression and found an increased expression of IL-1β in CHI3L1 KO cells compared to WT/sgCTR counterpart (Fig. 6e-f, Suppl. Fig. 4e, 5d-e). To validate whether IL-1β drives AQP4 expression in astrocytes, we treated astrocytes with GCM from U87-MG CHI3L1 KO with or without an IL-1β neutralizing antibody. We found that IL-1β neutralization diminished both AQP4 and NFκB expression (Fig. 6g-h) in those astrocytes.

## Discussion

In this study by exploring scRNAseq databases and bulk RNAseq data from two Polish glioma patient cohorts, we found upregulated expression of *CHI3L1* in malignant gliomas; the highest was found in GBMs compared to other cancers in the GEPIA database. Malignant cells were the predominant source of *CHI3L1* expression in GBMs. A positive correlation between genes from the MESlike1 signature and CHI3L1 protein in the Brain Protein Atlas suggests the link between *CHI3L1* expression and the mesenchymal phenotype of GBM. In a recently published study, Guetta-Terrier and colleagues [52] report that CHI3L1 is a driver of mesenchymal phenotype of glioma stem cells (GCS). This agrees with the presented RNAseq results where genes associated with a mesenchymal subtype are one of the most down-regulated DEGs along *CHI3L1* in CHI3L1 KO cells (Fig. 3g-h). U87-MG glioma cells used in this study are enriched in GSCs [53]. Our findings suggest that CHI3L1, apart from being a member of mesenchymal signature, might be a potential positive regulator of the mesenchymal signature genes. Increased expression of *CHI3L1* was found also in pilocytic astrocytomas (PA) which are non-invasive tumors [54] but the protein content of CHI3L1 has been reported low in PA [55]. This discrepancy might be due to the presence of mRNA-stabilizing HuR protein frequently up-regulated high-grade gliomas [56], where it drives excessive protein synthesis.

As CHI3L1 KO glioma cells formed significantly smaller tumors compared to WT cells, we conclude that CHI3L1 is required for tumor progression. This conclusion is in line with previous studies showing that the overexpression of CHI3L1 yielded larger tumors in recipient mice [57]. Interestingly, this phenomenon was only observed in immunocompetent mice as compared to SCID mice, which indicates that the immune microenvironment plays a pivotal role in the impact of CHI3L1 on tumor growth. The RNAseq results showed that genes associated with “DNA replication” were down-regulated in CHI3L1 KO cells, however, three independent cell proliferation assays including BrdU incorporation assay, cell dye dilution assay and Ki67 staining on tumor sections showed comparable proliferation of WT and CHI3L1 OK cells (Fig. 4g-j, Suppl. Fig. 3e). CRISPR/Cas9 protocol does not change the proliferation rate or metabolic activity of modified glioma cells as evidenced using sgCTR clones (Suppl. Fig. 2d, 4d).

As CHI3L1 depletion did not affect basal proliferation of glioma cells, we can attribute the reduced tumor growth in vivo to defective interactions of tumor cells with the TME. Transcriptomic analysis of CHI3L1 KO glioma cells revealed downregulation of groups of genes that pointed to several tumor supportive mechanisms being affected in KO cells: (1) regulators of ECM rearrangement; (2) markers of myeloid cells infiltration/reprograming; (3) modulators of neoangiogenesis. We verified several of those mechanisms in in vitro and in vivo GBM models and provided insights on their molecular underpinning.

We found significantly reduced MMP-2 expression and gelatinolytic activity in CHI3L1 KO cells. MMP-2 is a crucial ECM degrading enzyme, produced by glioma cells and activated through interactions with activated microglia supplying MT-1MMP (MMP14) [40]. We have previously reported that glioma invasion requires glioma-associated microglia that contribute to MMP2 activation [40]. Consistently, microglia-dependent invasion of CHI3L1 KO cells was reduced in comparison to invasion of WT cells (as evidenced with glioma-microglia co-cultures and Matrigel assay).

We have previously reported that tumor-derived SPP1/osteopontin, a multifunctional protein associated with the regulation of tumor invasion and immunosuppression in GBM, contributes to accumulation of microglia in gliomas [39, 53, 58]. Interestingly, we found a strong positive correlation between *CHI3L1* and *SPP1* expression in 14 primary cell lines established with human glioblastoma samples [27] (Suppl. Fig. 3f), which is in line with reports suggesting an interaction loop and strong dependency between CHI3L1 and SPP1 in glioblastoma [59]. We found that CHI3L1 KO cells have strongly diminished expression and secretion of SPP1. Reduced SPP1 production in CHI3L1 KO in vivo resulted in reduced accumulation of myeloid cells in the tumor margin in CHI3L1 KO tumors, as evidenced by IBA1 staining and quantification of IBA1 + cells on tumor sections. The lack of difference in myeloid cell infiltration within the tumor can be explained by a relatively late timepoint of tumor growth in our experiments (21 days post implantation). However, when IBA1 + cells were counted at the tumor margin (where typically microglia are abundant), differences in cell infiltration were detected. Moreover, myeloid cells infiltrating CHI3L1 KO tumors differed qualitatively from counterparts in WT tumors and expressed less SPP1 and less CD206, a scavenger receptor associated with immunosuppressive M2 phenotype of macrophages (Fig. 4f-g and k-l). This is in line with reports showing an inhibition of M2 macrophage polarization in cancer after a neutralization of CHI3L1 [60]. Another group of genes downregulated in CHI3L1 KO cells are genes associated with PD-1 and IL-10 signaling that are crucial signals for immunosuppression. *CSF1*, *CCL2* and *TGFB3* are important factors driving accumulation for myeloid cells in glioblastoma [61] and their downregulation might profoundly change the tumor microenvironment into less permissive for tumor growth [62, 63]. The genes associated with the mesenchymal signature were strongly down-regulated in CHI3L1 KO cells suggesting that CHI3L1 might be a positive regulator of some of the mesenchymal subtype genes. The MES signature has been linked to both myeloid cell accumulation and immunosuppression [64, 65].

**Fig. 7:**
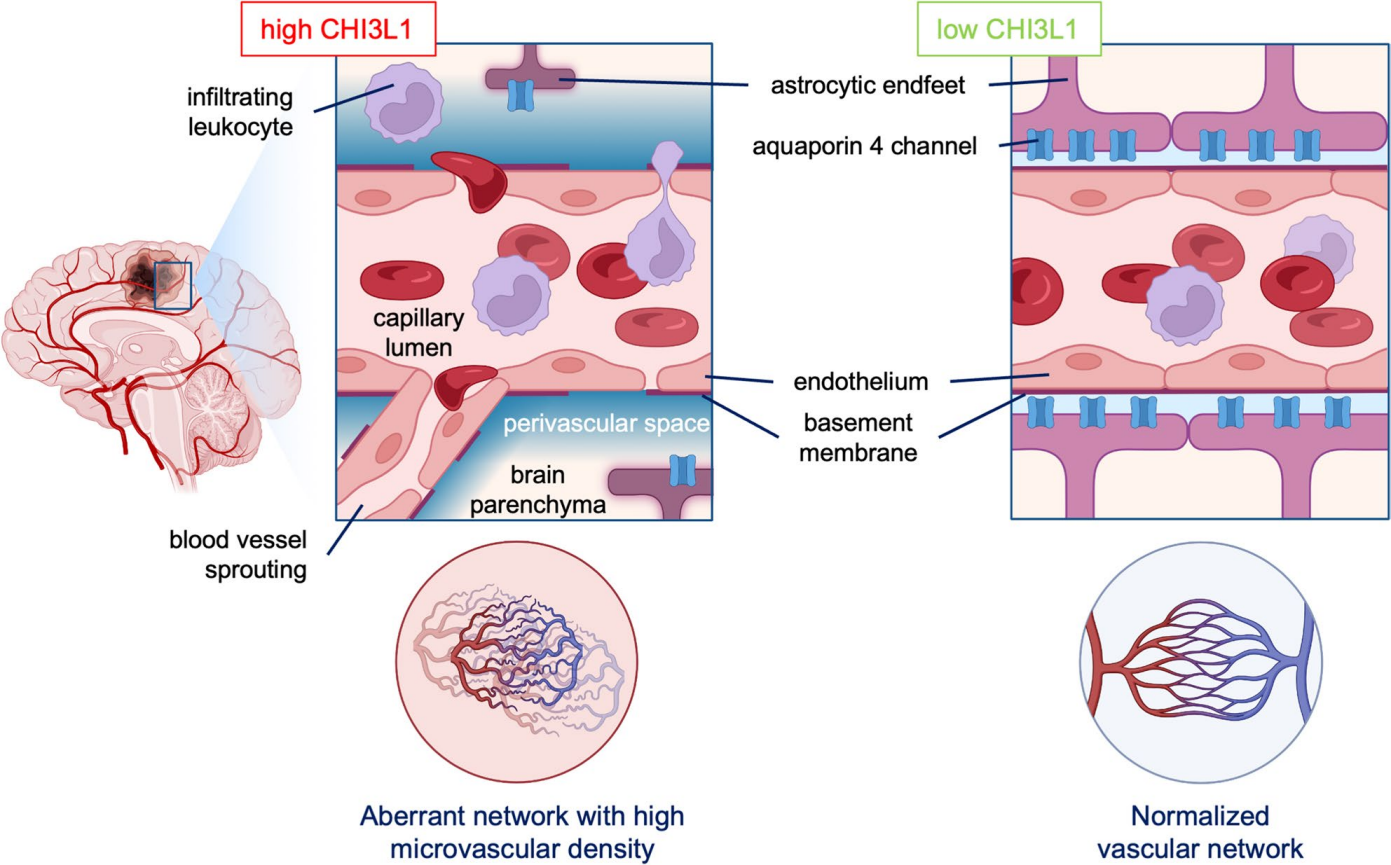
A proposed model showing the brain vasculature and perivascular space in WT and CHI3L1 KO gliomas. In tumors with high CHI3L1 production the number of large vessels decreases and the expanse of microvessels forms the disorganized vascular network. The vascular laminar basement membrane is fenestrated and thin. The aberrant vessels may facilitate trafficking of myeloid cell from the periphery. The blood-brain barrier is disrupted: astrocytes retract from vessels and the perivascular space is not sealed by an astrocytic endfeet with a crucial water channel AQP4. In CHI3L1 KO gliomas, the vascular network is normalized, less leaky and more efficient in carrying blood flow. Endothelial basement membrane is well structured and thick. The number of activated astrocytes is reduced, astrocytic endfeet cover the blood vessels and seal the perivascular space

The mechanisms described above could directly or indirectly affect tumor vasculature. Aberrant vasculature is a hallmark of GBMs which frequently have collapsed and disorganized vessels, which contributes to hypoxia and immunosuppression, and creates a specific TME [18, 66–68]. It is a serious predicament reducing the effectiveness of antitumor immunity and immunotherapy. Astrocytes contacting the vessels via astrocytic endfeet are displaced by glioblastoma cells, which disrupts the blood-brain barrier (BBB) and leads to a damaging ‘permeability and retention effect’ [69]. The delivery of drugs to the tumor remains a key challenge in the treatment of GBM [70]. Therefore, the normalization of vasculature is desirable for an effective anti-tumor activity in GBM. Previous studies in in vitro models or extracranial mouse models reported the impact of CHI3L1 on vasculature in GBM, mostly via regulation of VEGF signaling [16, 17, 71]. In this study, we demonstrate that CHI3L1 KO intracranial tumors present features of a normalized vasculature network, with a greater number of non-capillary vessels, reduced density of microvessels and elevated AQP4 expression at the astrocytic endfeet surrounding the vessels. Using immortalized astrocytes exposed to glioma-conditioned media we demonstrate that the lack of CHI3L1 augments AQP4 protein content in those cells. The restoration of AQP4 expression was associated with the activation of p38/MAPK pathway, as the inhibition of p38/MAPK pathway blocked this upregulation. However, there might be other mechanisms involved because the p38/MAPK pathway inhibitor was not sufficient to interfere with AQP4 expression in astrocytes co-cultured with CHI3L1 KO cells. While the exact mechanism between CHI3L1 and AQP4 expression remains elusive, we focused on the phenotype of glioma cells depleted of CHI3L1 to look for a mechanism linking the two proteins. We demonstrate that glioma cells depleted of CHI3L1 have upregulated IL-1β expression (Fig. 6e-f). IL-1β has been reported as a driver of AQP4 expression in murine astrocytes via NFκB [51]. We showed that the neutralization of IL-1β in CHI3L1 KO GCM diminished both AQP4 and NFκB expression in astrocytes. We propose IL-1β signaling through NFκB to be the driver of AQP4 expression in astrocytes treated with GCM. Notably, the inhibition of p38 was not sufficient to block AQP4 expression in astrocytes (Fig. 6d), while the inhibition of IL-1β was (Fig. 6h), which suggest a synergy of several pathways in the regulation of AQP4 with IL-1β signaling being the predominant regulation pathway. While the contribution of VEGF was not directly tested in this study, we have analyzed human datasets and found a positive correlation for VEGFA and CHI3L1 in the Ivy GAP database (Suppl. Fig. 3c). Measurements of mVEGF and mCHI3L1 concentration in blood of tumor-bearing mice in CHI3L1 KO group showed a strong positive correlation (Suppl. Fig. 3d) suggesting a synergistic action or interdependence of CHI3L1 and VEGF in regulating neoangiogenesis. A scheme in the Fig. 7 presents potential mechanisms driven by CHI3L1 in glioma vasculature.

In summary, we demonstrate that CHI3L1 depletion in glioma cells affects several mechanisms crucial for GBM progression: (1) it grossly affects transcriptional profile and reduces glioma cell invasion in vitro which might limit tumor growth in mice; (2) it results in normalization of tumor vasculature likely via upregulation Aquaporin 4 in p38/MAPK and IL-1β/NFκB-dependent manner; (3) it diminishes the infiltration of glioma-associated myeloid cells in CHI3L1 KO tumours due to reduced SPP1 expression in CHI3L1 KO cells. Our results demonstrate that CHI3L1 is an important therapeutic target in GBM and downregulating its expression or activity might have a multi-facet impact on various pathological processes crucial for the tumor progression.

### Limitations of the study

We were unable to explore all complex interactions between various cells and the mechanisms through which this interplay occurs resulting in halted tumor progression. We did not monitor the effects of host mouse CHI3L1 to the tumor presence, although the observed lack of correlation between tumor size and host mouse CHI3L1 serum concentration suggests that such effects are negligible.

We were unable to validate all RNAseq results. We did not perform an in-depth analysis of cell cycle arrest or stress-related DNA replication/repair mechanisms that could lead to down-regulation of genes in “DNA replication” pathway, although we performed three independent cell proliferation assays to address this issue and strengthen our reasoning.

Also, the normalization of the vasculature could happen through various mechanisms, and we evaluated only potential contribution of astrocytic aquaporin 4 and tumor derived VEGF. We cannot exclude the existence of dynamic interactions between myeloid cells and cells forming new vessels. The study would benefit from vessel permeability experiments with Evans blue.

Another limitation of this study is a small set of tested antibodies to dissect functional phenotypes of myeloid cells infiltrating CHI3L1 KO gliomas. We did not test the possibility of M1-like inflammatory macrophages targeting the CHI3L1 deficient glioma cells. More selective markers are required to define a functional phenotype of infiltrating cells.

## Supplementary Information

Supplementary Material 1: Figure 1. Ratio of *CHI3L1* expression in tumor versus normal tissue. Data from the GEPIA database. ACC adrenocortical carcinoma, BLCC bladder Urothelial Carcinoma, BRCA breast invasive carcinoma, CESC cervical squamous cell carcinoma and endocervical adenocarcinoma, CHOL cholangiocarcinoma, COAD colon adenocarcinoma, DLBC lymphoid neoplasm diffuse large b-cell lymphoma, ESCA esophageal carcinoma, GBM glioblastoma, HNSC head and neck squamous cell carcinoma, KICH kidney chromophobe, KIRC kidney renal clear cell carcinoma, KIRP kidney renal papillary cell carcinoma, LAML acute myeloid leukemia, LGG brain lower grade glioma, LIHC liver hepatocellular carcinoma, LUAD lung adenocarcinoma, LUSC lung squamous cell carcinoma, MESO mesothelioma, OV ovarian serous cystadenocarcinoma, PAAD pancreatic adenocarcinoma, PCPG pheochromocytoma and paraganglioma, PRAD prostate adenocarcinoma, READ rectum adenocarcinoma, SARC sarcoma, SKCM skin cutaneous melanoma, STAD stomach adenocarcinoma, TGCT testicular germ cell tumors, THCA thyroid carcinoma, THYM thymoma, UCEC uterine corpus endometrial Carcinoma, UCS uterine carcinosarcoma, UVM uveal melanoma.

Supplementary Material 2: Figure 2. a. Gating strategy for the flow cytometric sort of U87-MG-RFP transfected with pCMV-Cas9-GFP plasmid. b-c. Validation of *CHI3L1* knock-out in selected candidate clones. b. RT-qPCR analysis of CHI3L1 expression using 2 sets of primers. CHI3L1 expression given as % of WT control. c. ELISA of cell culture supernatants of sgCHI3L1 clones. CHI3L1 concentration given as % of WT control. Red lines indicate the level in WT reference. d. BrdU incorporation assay for U87-MG-RFP cells transfected with control non-targeting gRNA (sgCTR). CRISPR/Cas9 engineering pipeline does not change the proliferation rate of U87-MG-RFP clones. e. Analysis of *CHI3L1* expression in NHA, U251, U87-MG and WG9 by RT-qPCR

Supplementary Material 3: Figure 3. a. Correlation analysis for VEGFA and CHI3L1 expression in Ivy GAP RNAseq database. b. Correlation analysis for mVEGF and mCHI3L1 in the sera of tumor-bearing mice. c. Immunofluorescent staining for Ki67, a mitosis/proliferation marker, in green. Scale bar represents 150 μm. Enlarged regions of interest depicting nuclear staining are presented below. d. Linear regression analysis of *CHI3L1* and *SPP1* mRNA expression in 14 human primary cell lines described elsewhere [27]. 

Supplementary Material 4: Figure 4. a. Gating strategy for the flow cytometric sort of U251 transfected with pCMV-Cas9-GFP plasmid. Percent of positive GFP signal is presented. b-c. Validation of *CHI3L1* knock-out in selected sgCTR and CHI3L1 KO clones. b. RT-qPCR analysis of *CHI3L1* expression. * *P* ≤ 0.05. c. ELISA of cell culture supernatants of sgCTR and CHI3L1 KO clones. ** *P* ≤ 0.01. d. MTT metabolic activity assay for selected sgCTR and CHI3L1 KO clones. e Gene expression analysis for MMP2 and IL-1β in CHI3L1 KO cells. Black line represents gene expression for sgCTR. * *P* ≤ 0.05, ** *P* ≤ 0.01. f. Matrigel invasion assay for U251 sgCTR and CHI3L1 KO cells. Black bars represent baseline invasiveness of glioma cells; grey and red dots/bars represent invasiveness of sgCTR and CHI3L1 KO cells when co-cultured with HM-SV40 human microglial cells; mean ± SD is presented. * *P* ≤ 0.05.

Supplementary Material 5: Figure 5. a. Gating strategy for the flow cytometric sort of WG9 transfected with pCMV-Cas9-GFP plasmid. Percent of positive GFP signal is presented. b-c. Validation of *CHI3L1* knock-out in selected sgCTR and CHI3L1 KO clones. b. RT-qPCR analysis of *CHI3L1* expression. * *P* ≤ 0.05. c. ELISA of cell culture supernatants of sgCTR and CHI3L1 KO clones. ** *P* ≤ 0.01. d. Gene expression analysis for MMP2 and IL-1β in CHI3L1 KO cells. Black line represents gene expression for sgCTR. *P ≤ 0.05, ** *P* ≤ 0.01. e Matrigel invasion assay for WG9 sgCTR and CHI3L1 KO cells. Black bars represent baseline invasiveness of glioma cells; grey and red dots/bars represent invasiveness of sgCTR and CHI3L1 KO cells when co-cultured with HM-SV40 human microglial cells; mean ± SD is presented.

Supplementary Material 6.

## Data Availability

RNAseq data are available in the NIH GEO database with the accession number GSE231816. All other data supporting the findings of this study are available within the manuscript or the supplementary information or from the corresponding author upon a reasonable request. The tumor types expression data are publicly available at [http://gepia.cancer-pku.cn](http://gepia.cancer-pku.cn). The single-cell RNA expression data are publicly available at [https://singlecell.broadinstitute.org/single/_cell/study/SCP393/single-cell-rna-seq-of-adult-and-pediatric-glioblastom](https://singlecell.broadinstitute.org/single_cell/study/SCP393/single-cell-rna-seq-of-adult-and-pediatric-glioblastom). The protein atlas data are not publicly available but are available upon reasonable request from the corresponding author.
