# Supplementary figures and images for: Chitinase-3-like protein 1 depletion in glioma cells alters tumor microenvironment and normalizes neovasculature in human glioma xenografts
Source: Cell Commun Signal. 2026 Jan 15;24:103. doi: 10.1186/s12964-025-02636-8 (PMC12892814; doi:10.1186/s12964-025-02636-8)

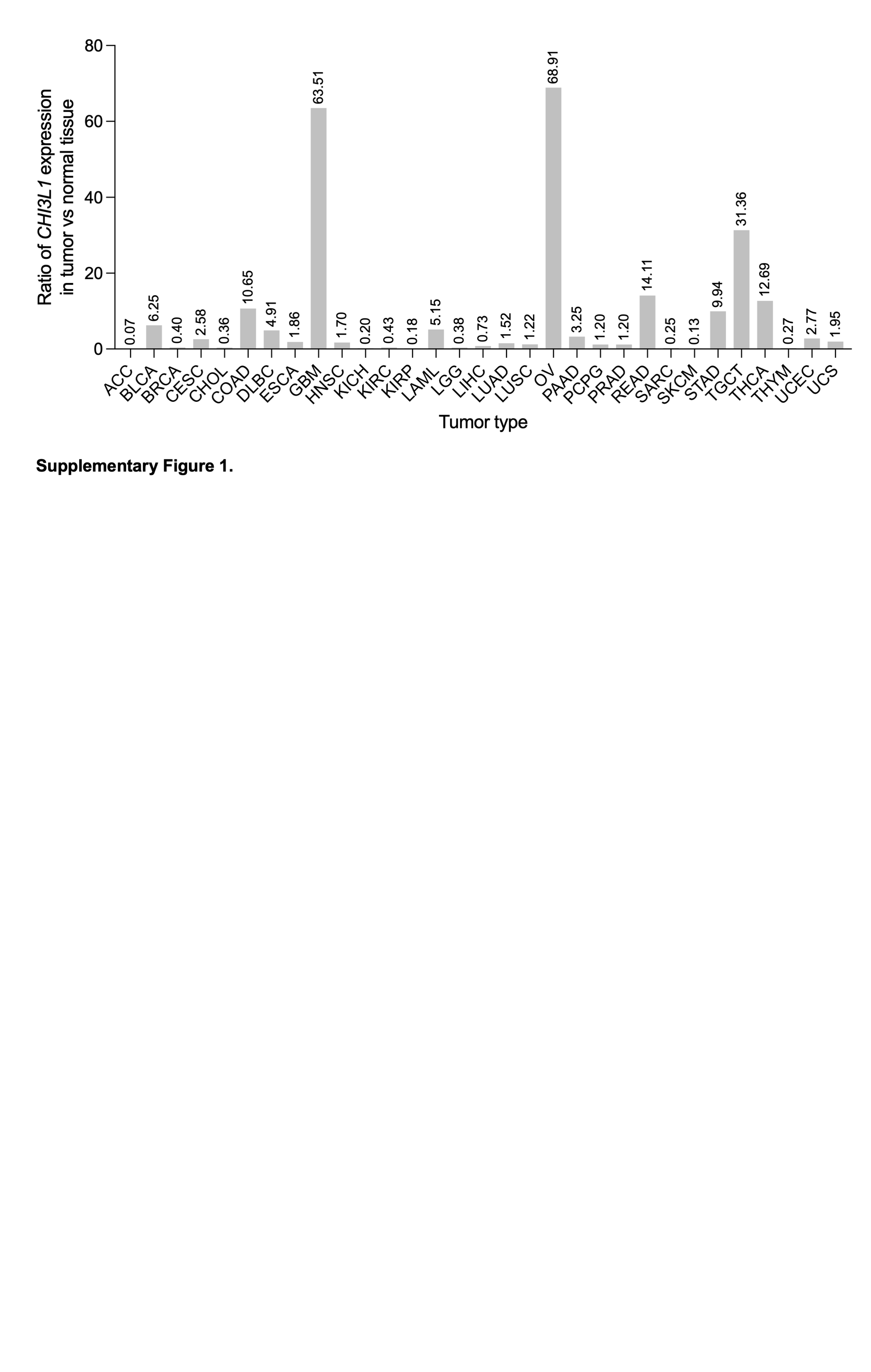

Supplement: Supplementary file 1 — Supplementary Material 1: Figure 1. Ratio of CHI3L1 expression in tumor versus normal tissue. Data from the GEPIA database. ACC adrenocortical carcinoma, BLCC bladder Urothelial Carcinoma, BRCA breast invasive carcinoma, CESC cervical squamous cell carcinoma and endocervical adenocarcinoma, CHOL cholangiocarcinoma, COAD colon adenocarcinoma, DLBC lymphoid neoplasm diffuse large b-cell lymphoma, ESCA esophageal carcinoma, GBM glioblastoma, HNSC head and neck squamous cell carcinoma, KICH kidney chromophobe, KIRC kidney renal clear cell carcinoma, KIRP kidney renal papillary cell carcinoma, LAML acute myeloid leukemia, LGG brain lower grade glioma, LIHC liver hepatocellular carcinoma, LUAD lung adenocarcinoma, LUSC lung squamous cell carcinoma, MESO mesothelioma, OV ovarian serous cystadenocarcinoma, PAAD pancreatic adenocarcinoma, PCPG pheochromocytoma and paraganglioma, PRAD prostate adenocarcinoma, READ rectum adenocarcinoma, SARC sarcoma, SKCM skin cutaneous melanoma, STAD stomach adenocarcinoma, TGCT testicular germ cell tumors, THCA thyroid carcinoma, THYM thymoma, UCEC uterine corpus endometrial Carcinoma, UCS uterine carcinosarcoma, UVM uveal melanoma. [file 12964_2025_2636_MOESM1_ESM.tiff]

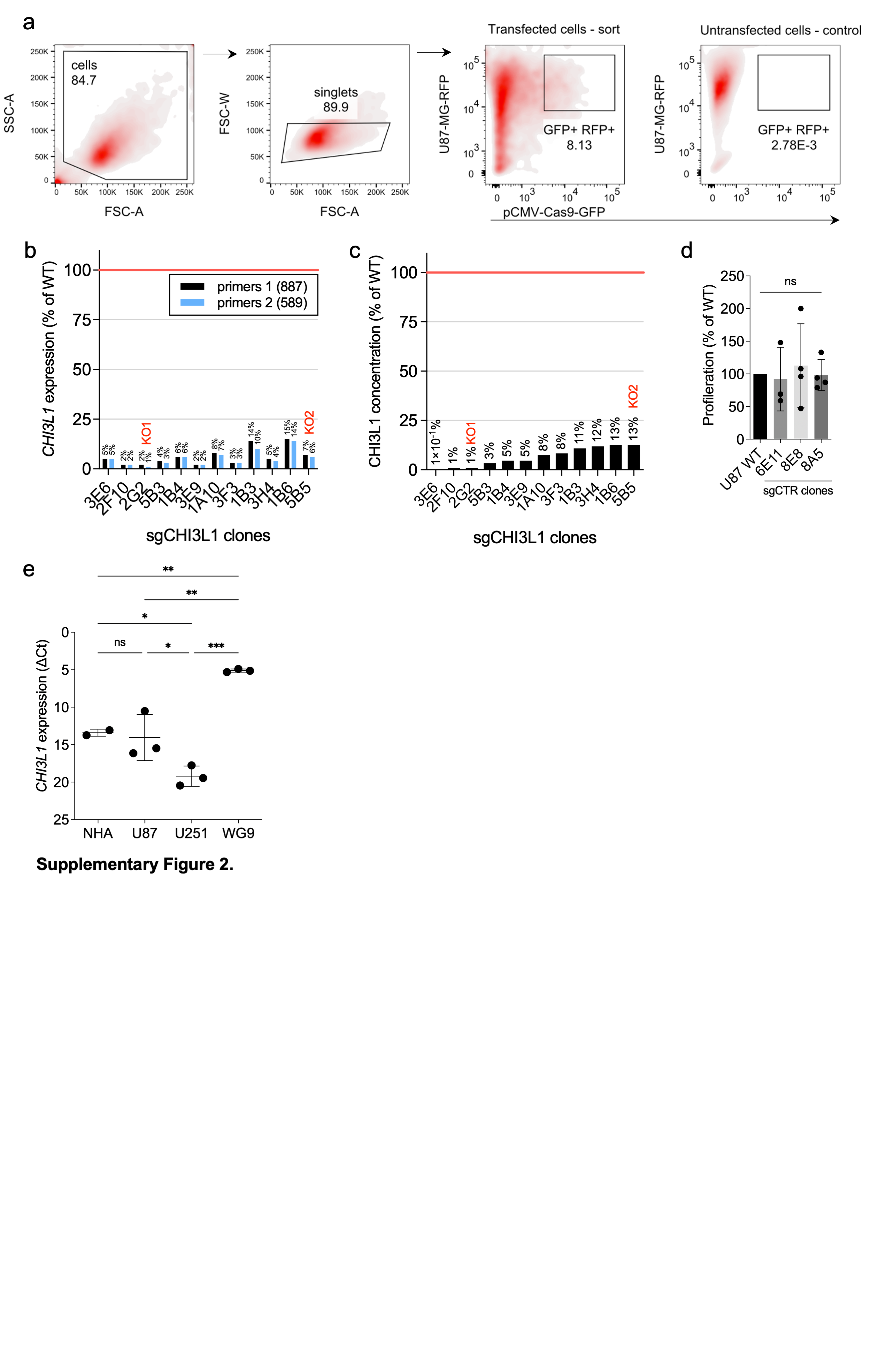

Supplement: Supplementary file 2 — Supplementary Material 2: Figure 2. a. Gating strategy for the flow cytometric sort of U87-MG-RFP transfected with pCMV-Cas9-GFP plasmid. b-c. Validation of CHI3L1 knock-out in selected candidate clones. b. RT-qPCR analysis of CHI3L1 expression using 2 sets of primers. CHI3L1 expression given as % of WT control. c. ELISA of cell culture supernatants of sgCHI3L1 clones. CHI3L1 concentration given as % of WT control. Red lines indicate the level in WT reference. d. BrdU incorporation assay for U87-MG-RFP cells transfected with control non-targeting gRNA (sgCTR). CRISPR/Cas9 engineering pipeline does not change the proliferation rate of U87-MG-RFP clones. e. Analysis of CHI3L1 expression in NHA, U251, U87-MG and WG9 by RT-qPCR [file 12964_2025_2636_MOESM2_ESM.tiff]

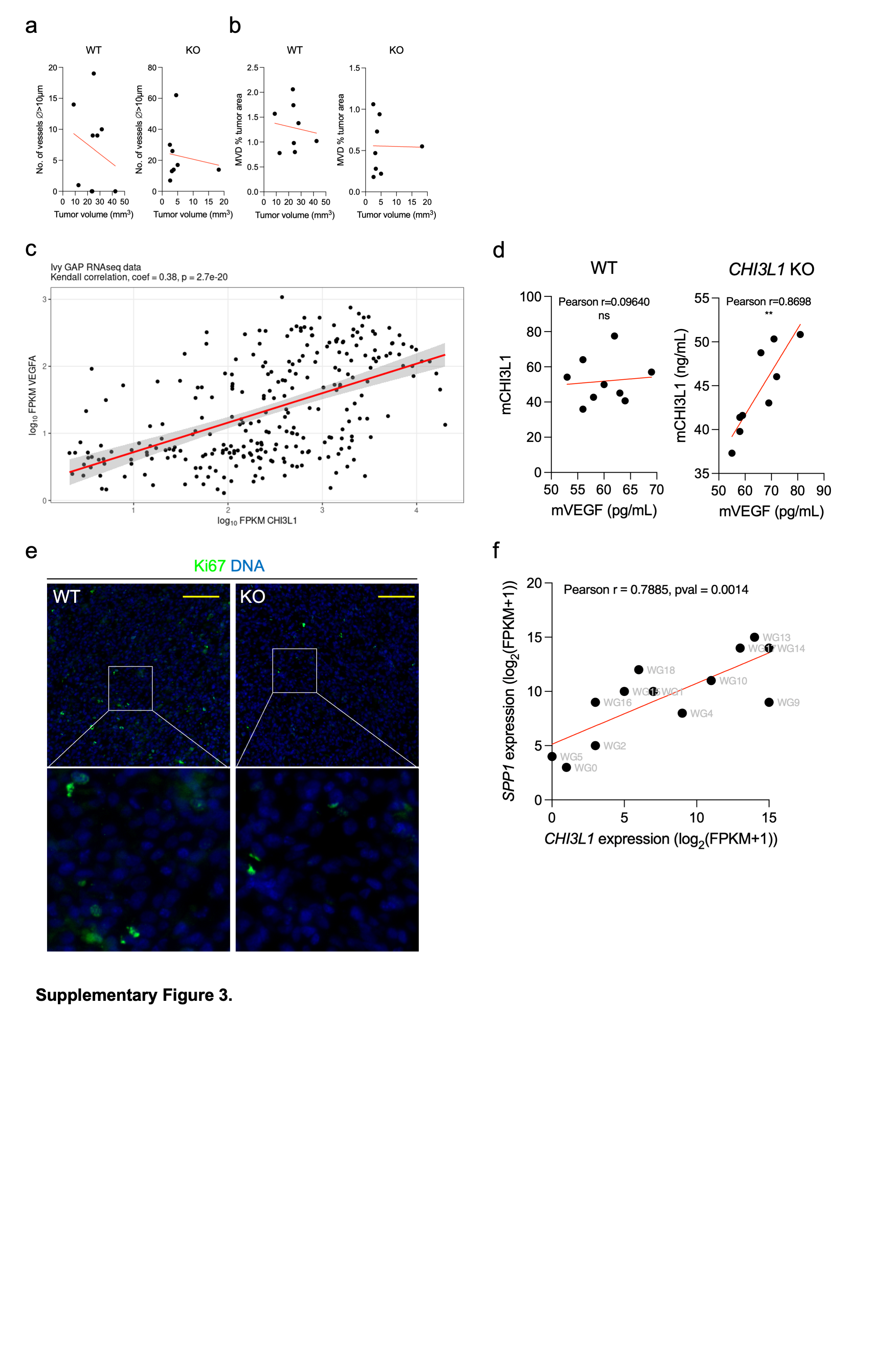

Supplement: Supplementary file 3 — Supplementary Material 3: Figure 3. a. Correlation analysis for VEGFA and CHI3L1 expression in Ivy GAP RNAseq database. b. Correlation analysis for mVEGF and mCHI3L1 in the sera of tumor-bearing mice. c. Immunofluorescent staining for Ki67, a mitosis/proliferation marker, in green. Scale bar represents 150 μm. Enlarged regions of interest depicting nuclear staining are presented below. d. Linear regression analysis of CHI3L1 and SPP1 mRNA expression in 14 human primary cell lines described elsewhere [27]. [file 12964_2025_2636_MOESM3_ESM.tiff]

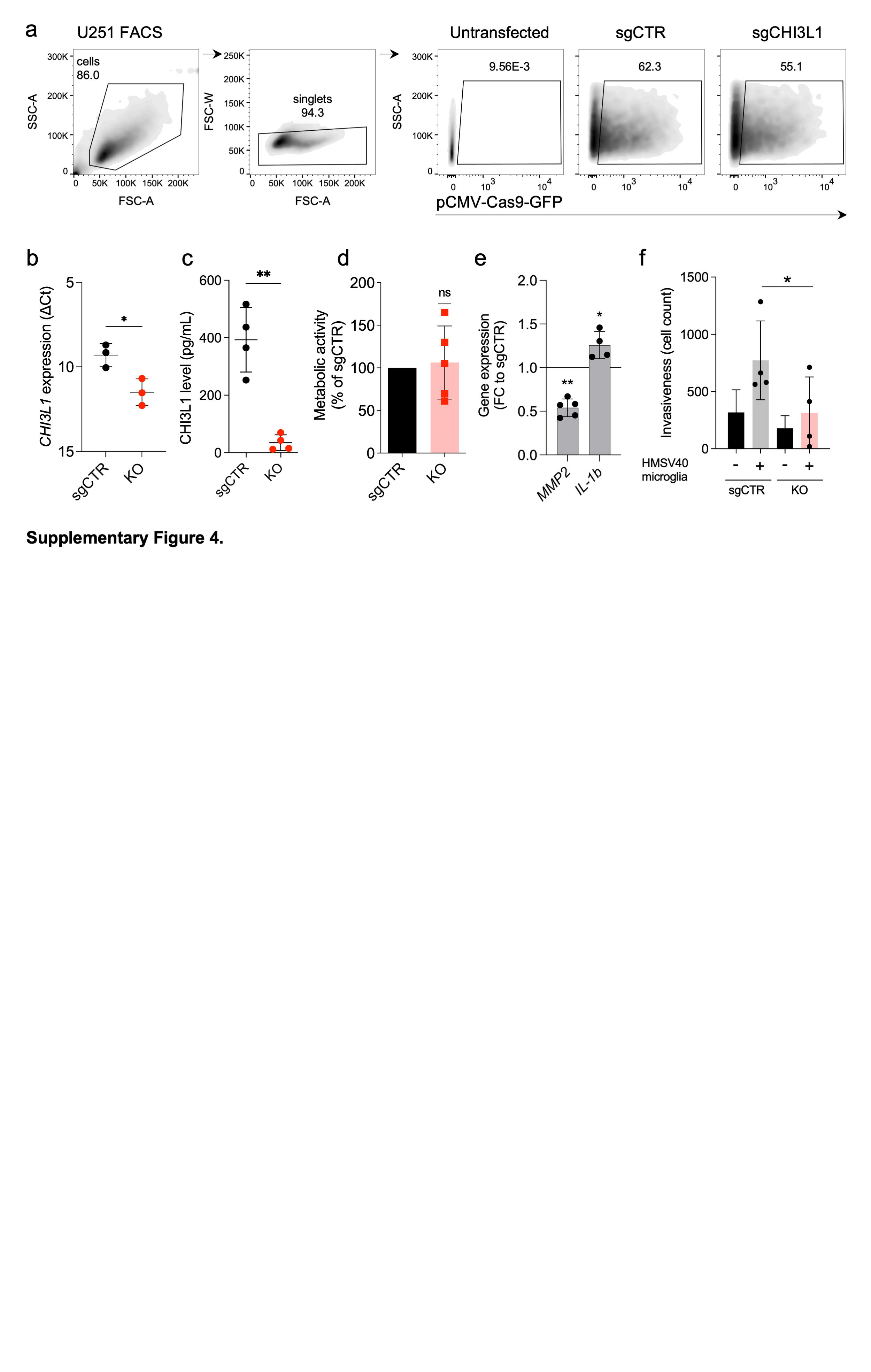

Supplement: Supplementary file 4 — Supplementary Material 4: Figure 4. a. Gating strategy for the flow cytometric sort of U251 transfected with pCMV-Cas9-GFP plasmid. Percent of positive GFP signal is presented. b-c. Validation of CHI3L1 knock-out in selected sgCTR and CHI3L1 KO clones. b. RT-qPCR analysis of CHI3L1 expression. * P ≤ 0.05. c. ELISA of cell culture supernatants of sgCTR and CHI3L1 KO clones. ** P ≤ 0.01. d. MTT metabolic activity assay for selected sgCTR and CHI3L1 KO clones. e Gene expression analysis for MMP2 and IL-1β in CHI3L1 KO cells. Black line represents gene expression for sgCTR. * P ≤ 0.05, ** P ≤ 0.01. f. Matrigel invasion assay for U251 sgCTR and CHI3L1 KO cells. Black bars represent baseline invasiveness of glioma cells; grey and red dots/bars represent invasiveness of sgCTR and CHI3L1 KO cells when co-cultured with HM-SV40 human microglial cells; mean ± SD is presented. * P ≤ 0.05. [file 12964_2025_2636_MOESM4_ESM.tiff]

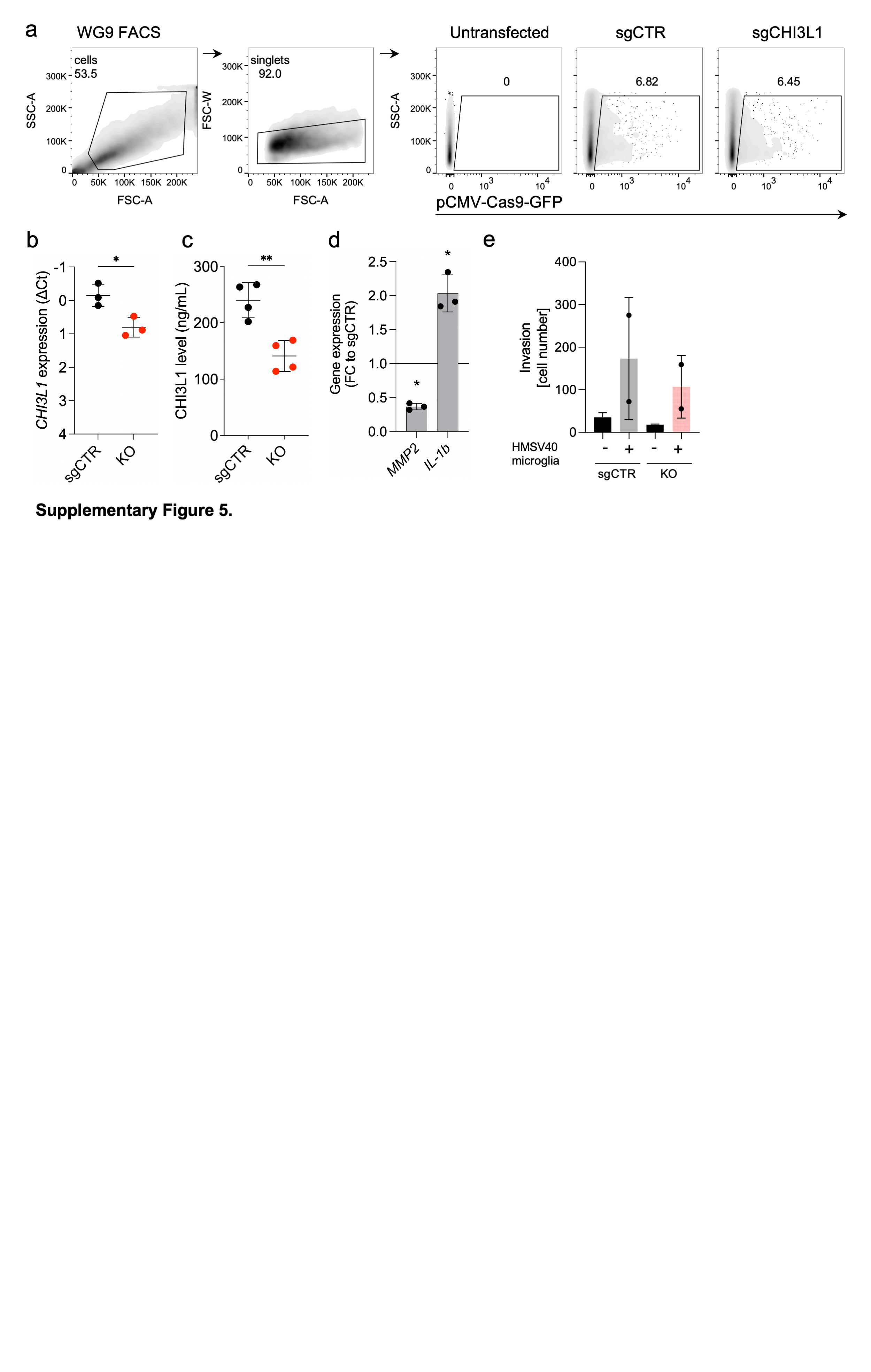

Supplement: Supplementary file 5 — Supplementary Material 5: Figure 5. a. Gating strategy for the flow cytometric sort of WG9 transfected with pCMV-Cas9-GFP plasmid. Percent of positive GFP signal is presented. b-c. Validation of CHI3L1 knock-out in selected sgCTR and CHI3L1 KO clones. b. RT-qPCR analysis of CHI3L1 expression. * P ≤ 0.05. c. ELISA of cell culture supernatants of sgCTR and CHI3L1 KO clones. ** P ≤ 0.01. d. Gene expression analysis for MMP2 and IL-1β in CHI3L1 KO cells. Black line represents gene expression for sgCTR. *P ≤ 0.05, ** P ≤ 0.01. e Matrigel invasion assay for WG9 sgCTR and CHI3L1 KO cells. Black bars represent baseline invasiveness of glioma cells; grey and red dots/bars represent invasiveness of sgCTR and CHI3L1 KO cells when co-cultured with HM-SV40 human microglial cells; mean ± SD is presented. [file 12964_2025_2636_MOESM5_ESM.tiff]
