# Supplementary material for: Chitinase-3-like protein 1 depletion in glioma cells alters tumor microenvironment and normalizes neovasculature in human glioma xenografts
Source: Cell Commun Signal. 2026 Jan 15;24:103. doi: 10.1186/s12964-025-02636-8 (PMC12892814; doi:10.1186/s12964-025-02636-8)

Fig. 4b

Cropped

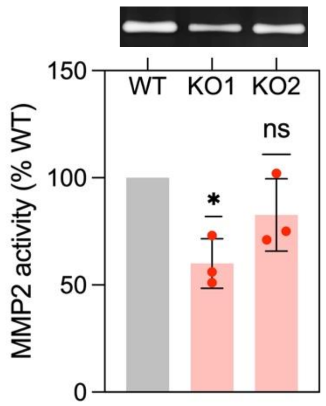

Uncropped

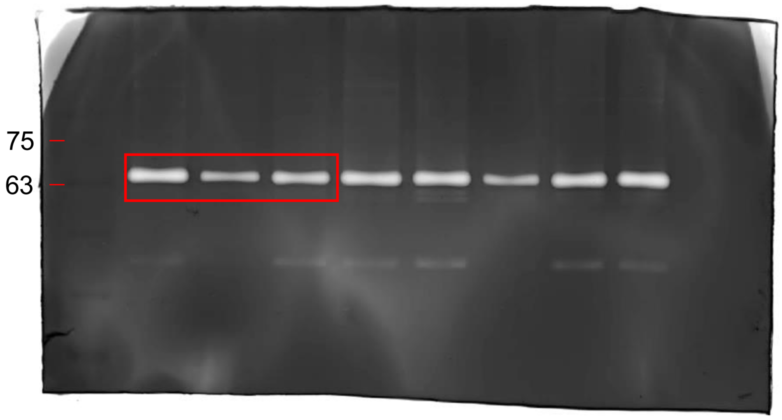

Fig. 6a

Cropped

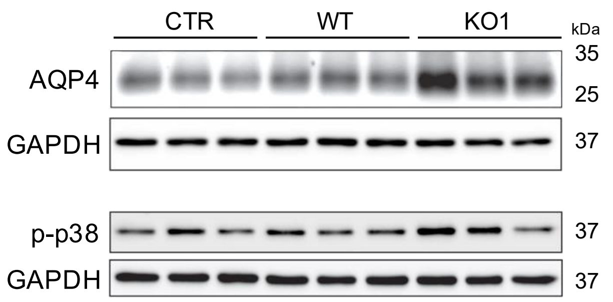

Uncropped

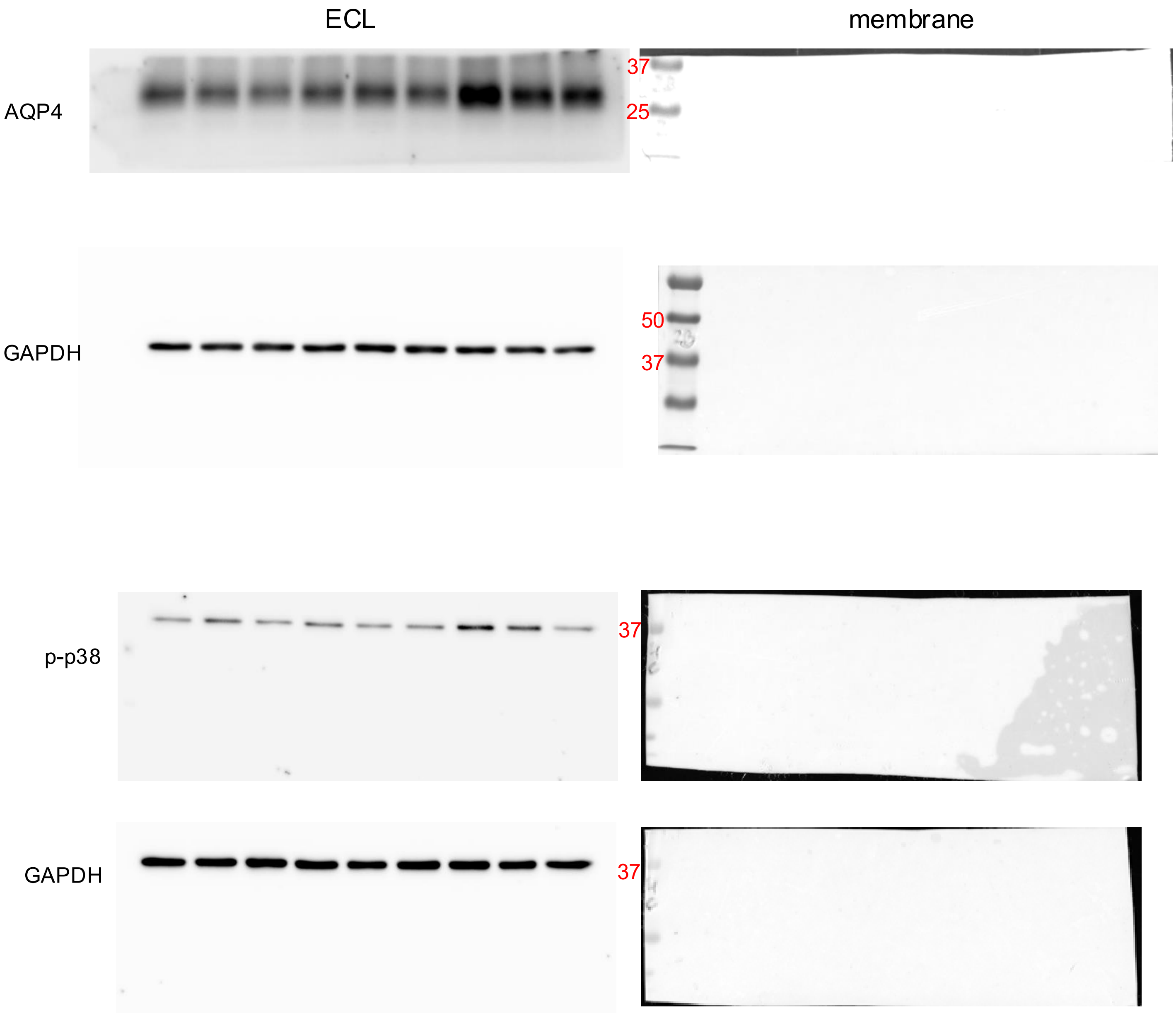

Fig. 6c

Cropped

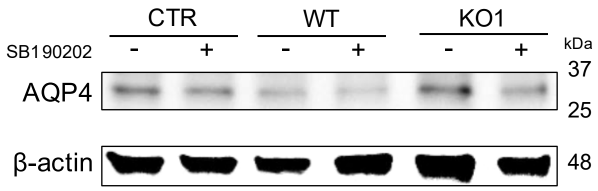

Uncropped

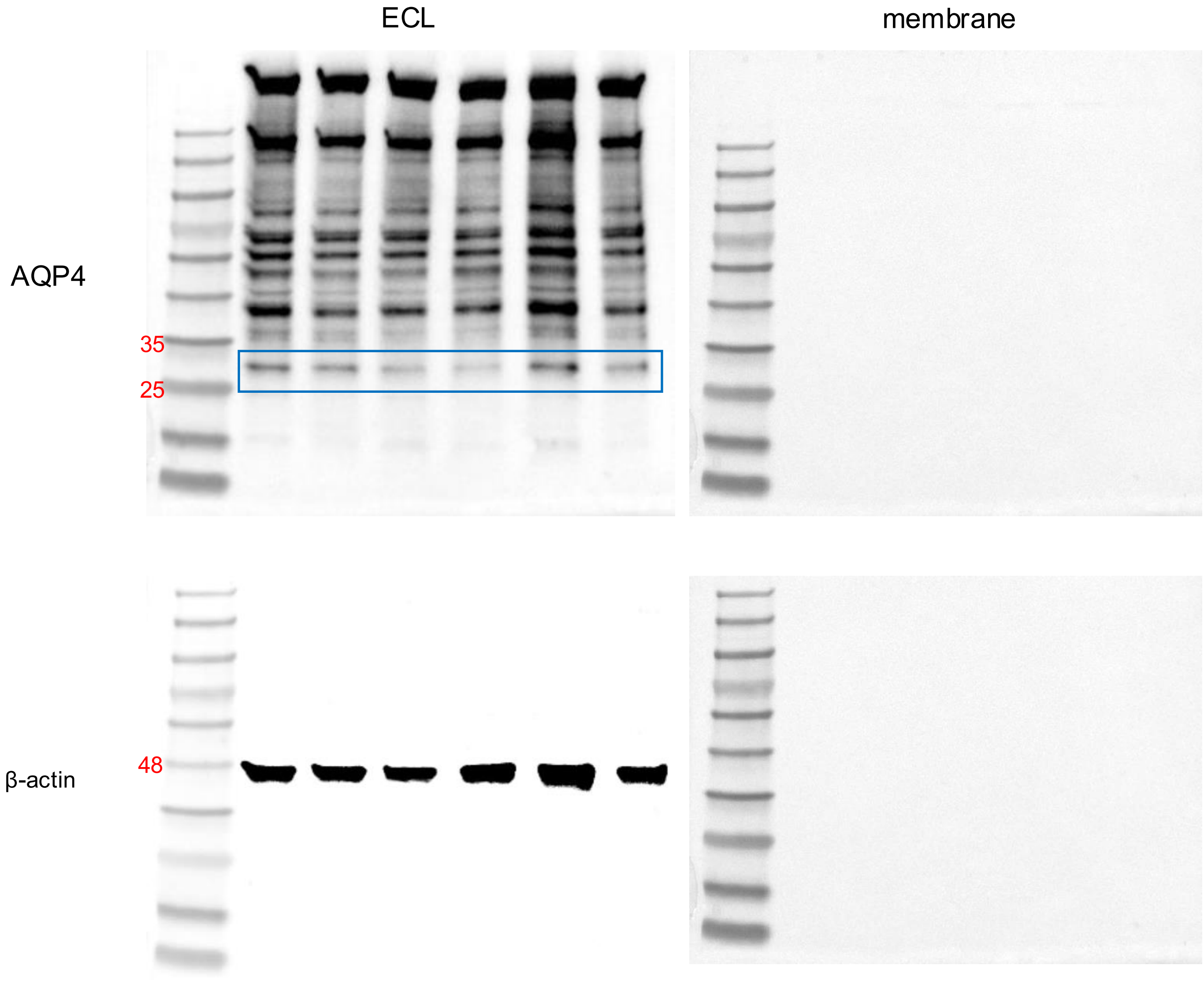

Fig. 6g

Cropped

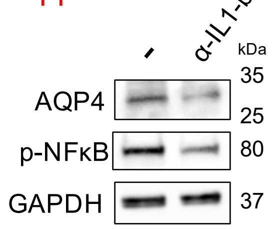

Uncropped

ECL

membrane

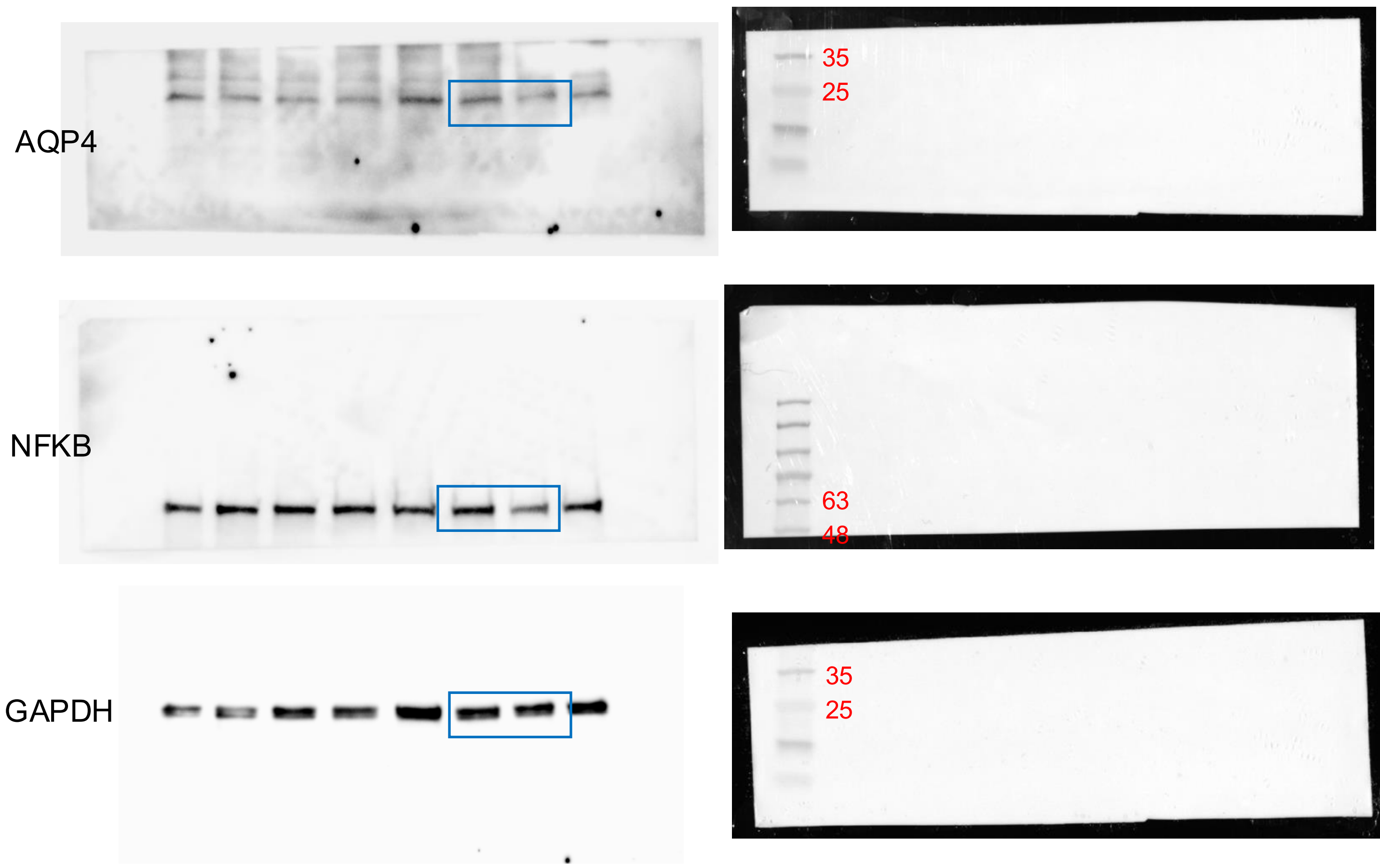

Supplement: Supplementary file 6 — Supplementary Material 6. [file 12964_2025_2636_MOESM6_ESM.pdf]
